# Correcting fake news headlines after repeated exposure: memory and belief accuracy in younger and older adults

**DOI:** 10.1186/s41235-024-00585-3

**Published:** 2024-08-26

**Authors:** Paige L. Kemp, Vanessa M. Loaiza, Colleen M. Kelley, Christopher N. Wahlheim

**Affiliations:** 1 Department of Psychology, University of North Carolina at Greensboro, 296 Eberhart Building, P. O. Box 26170, Greensboro, NC 27402-6170 USA; 2 School of Psychology, University of Sheffield, South Yorkshire, S1 2LT England; 3 Department of Psychology, Florida State University, Tallahassee, FL 32306 USA

**Keywords:** Fake news, Misinformation corrections, Memory updating, Beliefs, Cognitive aging

## Abstract

The efficacy of fake news corrections in improving memory and belief accuracy may depend on how often adults see false information before it is corrected. Two experiments tested the competing predictions that repeating fake news before corrections will either impair or improve memory and belief accuracy. These experiments also examined whether fake news exposure effects would differ for younger and older adults due to age-related differences in the recollection of contextual details. Younger and older adults read real and fake news headlines that appeared once or thrice. Next, they identified fake news corrections among real news headlines. Later, recognition and cued recall tests assessed memory for real news, fake news, if corrections occurred, and beliefs in retrieved details. Repeating fake news increased detection and remembering of corrections, correct real news retrieval, and erroneous fake news retrieval. No age differences emerged for detection of corrections, but younger adults remembered corrections better than older adults. At test, correct fake news retrieval for earlier-detected corrections was associated with better real news retrieval. This benefit did not differ between age groups in recognition but was greater for younger than older adults in cued recall. When detected corrections were not remembered at test, repeated fake news increased memory errors. Overall, both age groups believed correctly retrieved real news more than erroneously retrieved fake news to a similar degree. These findings suggest that fake news repetition effects on subsequent memory accuracy depended on age differences in recollection-based retrieval of fake news and that it was corrected.

## Significance statement

Fake news exposure can negatively impact memories and beliefs. To combat such exposure, we must understand how corrections mitigate these effects. Identifying differences between younger and older adults is important because age-related cognitive changes could erode the accuracy of memory for and beliefs in everyday news. One view proposes that more fake news exposure before corrections will improve memory and belief accuracy for younger adults and impair such accuracy for older adults. A competing view proposes that more frequent exposure to fake news will improve correction efficacy for both younger and older adults when corrections can later be remembered. We asked younger and older adults to read fake news headlines that appeared once or thrice before reading corrections. We then asked participants to retrieve real news details and rate the extent to which the retrieved details were accurate. More fake news exposure did not impair memory or belief accuracy for either age group. In fact, more fake news exposure improved younger and older adults’ overall memory for real news having corrected fake news. However, repeated fake news did have negative consequences for memory accuracy when participants could not remember that fake news was corrected. Collectively, these findings suggest that more exposure to fake news could improve memory for its content and accuracy after it is corrected. This may be helpful because it helps people discern true from false details. However, the costs of fake news exposure when corrections cannot be remembered must also be considered because this will occur more often over time and for people with memory impairments.

## Introduction

Exposure to misinformation on the internet can influence beliefs that negatively affect everyday decisions for adults of all ages. Underscoring this, beliefs in COVID-19 misinformation were shown to be associated with reduced self-reported intentions to vaccinate and follow health guidelines (Loomba et al., 2021; Roozenbeek et al., 2020). The rapid dissemination of misinformation across multiple internet platforms may result in people being repeatedly exposed to it before fact-checkers can issue corrections. This may lead to inaccurate beliefs and memories that elevate the risk of misguided decisions. Such exposure may have more detrimental consequences for older than younger adults because older adults experience poorer recollection of contextual details (e.g., Jennings & Jacoby, 1993), which may include veracity information. This creates a fundamental issue in society—we need methods for mitigating the negative effects of misinformation exposure that consider age-related memory differences. This requires identifying the mechanisms underlying the effects of repeated misinformation exposure on memory and beliefs in younger and older adults.

Repeated exposure to misinformation before corrections can negatively or positively affect memory for true details and the accuracy of beliefs in younger adults. Repeating misinformation during corrections sometimes leads to greater beliefs in misinformation (Nyhan et al., 2014), while in other cases, it diminishes the influence of misinformation on inferential reasoning (Ecker et al., 2017). Also, the negative effects of misinformation exposure during corrections are more likely when people forget that the misinformation was corrected (Kemp et al., 2022a, 2022b; Wahlheim et al., 2020). Prior studies have explored the consequences of repeating misinformation before corrections in narrative-based text comprehension paradigms with fictional scenarios (Ecker et al., 2011, 2017). However, none have examined how repeating fake news headlines from the internet before fact-check verified corrections affects subsequent retrieval of true details and beliefs in those details. Addressing this gap is crucial for determining when exposure to misinformation before corrections impairs or improves memory for details that may guide beliefs and decisions.

We addressed this issue here in two experiments that manipulated misinformation exposure before corrections by varying the number of repetitions of fake news headlines from the internet. We examined potential age differences in how people could detect unlabeled misinformation corrections and subsequently remember both fake and real news details as well as if those details were accurate. Repeated exposure to fake news may be particularly problematic for older adults because their reduced recollection of associations (for a review, see Park & Festini, 2017) may undermine their ability to remember differences between fake and real news. Understanding the consequences of repeating fake news before corrections in older adults is also needed because of their increased engagement with fake news on social media platforms (Grinberg et al., 2019; Guess et al., 2019), which may reflect limited digital media literacy in some circumstances (Brashier & Schacter, 2020). It is also concerning that older adults share fake news with greater personal relevance (i.e., health misinformation), even when prompted to consider the information’s accuracy (Zhou et al., 2023).

We motivate the present study below by summarizing select findings and theories from the literatures on the continued influence of misinformation and age-related differences in the recollection of associative information. Researchers have often examined the consequences of misinformation exposure using a narrative-based paradigm in which participants read an unfolding fictitious event (Johnson & Seifert, 1994; Wilkes & Leatherbarrow, 1988). The event includes a specific misinformation detail that is later corrected or not. Correction efficacy is then assessed with inferential reasoning questions that evaluate the influence of the misinformation detail. People consistently continue to rely on the misinformation in their inferential reasoning even when they remember that a correction was issued earlier (for a review, see Lewandowsky et al., 2012). This continued influence effect is robust, as it has been replicated using various materials (e.g., news reports and myths) in laboratory and online settings (Desai & Reimers, 2019; Ecker et al., 2011; Rich & Zaragoza, 2016, 2020). Corrections vary in their efficacy—being more effective when coherent, congruent with existing beliefs, and from credible sources—but they do not entirely eliminate the influence of misinformation (for a review and meta-analysis, see Walter & Tukachinsky, 2020).

Several theories have been invoked to account for the continued influence effect (for a review, see Lewandowsky et al., 2012). Most germane are dual-process and source memory accounts. Dual-process theories propose that retrieval can be based on recollection including contextual details, such as veracity and source, or acontextual familiarity that varies in strength (Ayers & Reder, 1998; Jacoby, 1991; Yonelinas, 2002). Accordingly, after misinformation and corrections are encoded, they co-exist in memory and compete for activation at retrieval. Repeating misinformation increases its familiarity and ease of processing (Schwarz et al., 2007), and therefore its potential to influence subsequent memory and reasoning. When contextual details are not recollected, the misinformation made familiar by repetition becomes a more attractive response candidate. Similarly, the source monitoring framework proposes that people can separately remember both content and the source from which it originated (Johnson et al., 1993). Consequently, remembering the content without the source, especially for familiar misinformation, can lead to memory errors confusing false with true information. These views are somewhat compatible with the finding that misinformation repetitions increase the continued influence effect (cf. Ecker et al., 2011).

Repeating misinformation during corrections can also increase familiarity and erroneous beliefs in naturalistic tasks other than the narrative-based paradigm. In a study employing a myth vs. fact message frame, participants read a flyer juxtaposing myths and facts associated with the flu vaccine (Skurnik et al., 2007; as cited in Schwarz et al., 2007). Beliefs were assessed immediately or after 30 min by requiring participants to identify whether statements were myths or facts. Performance was near perfect on an immediate test. But, after the delay, participants misremembered many myths as being true and expressed more negative attitudes toward the flu vaccine relative to participants who had not seen the flyer. In line with dual-process theories, these effects were attributed to poorer recollection after a delay and a stronger influence of myth familiarity resulting from myths appearing with facts (also see, Begg et al., 1992; Skurnik et al., 2005). Other studies support this view by showing that repeating misinformation with corrections can decrease the accuracy of beliefs (Autry & Duarte, 2021; Nyhan et al., 2014; Peter & Koch, 2016; Pluviano et al., 2017, 2019). However, this is not always the case (see Prike et al., 2023). The available literature suggests that more exposure to everyday misinformation, such as fake news headlines, can lead it to interfere with memory for corrective details, but only under specific circumstances.

Although some findings suggest that repeating misinformation creates the risk that familiarity will backfire, other research casts doubt on the robustness of this effect. A meta-analysis of familiarity backfire indicates that the effect often results from design artifacts, such as unreliable measurement and underpowered studies (Swire-Thompson et al., 2020). Additionally, studies using misinformation correction paradigms show that misinformation reminders reduce the influence of misinformation. In the narrative-based paradigm, reminders with corrective details reduced misinformation reliance on inferential reasoning (Ecker et al., 2017). This reduction was attributed to reminders promoting the co-activation of false and true information that improved encoding of the details and their conflict. This aligns with research indicating that knowledge revision is improved when conflicting details are detected (Kendeou et al., 2014, 2019; Stadtler et al., 2013).

Building on this view, research using news headlines from the internet has shown that exposure to real news that corrects fake news can improve memory and belief accuracy more when fake news reminders precede real news corrections (Kemp et al., 2022b; Wahlheim et al., 2020). This work showed that reminder effects reflect increased salience of conflicting details and improved recollection of their relationship. Further, conditional analyses suggested that reminders facilitated associative encoding and subsequent recollection of real news correcting fake news. However, the familiarity of fake news did lead to intrusion errors and less accurate beliefs when participants could not recollect that fake news had been corrected. These findings suggest that misinformation reminders during new learning can effectively counteract interference when detected detail changes promote later recollection of those changes (for a review, see Wahlheim et al., 2021). From this perspective, fake news reminders can enhance recollection by promoting integrative encoding but also increase familiarity-based errors for other items. Consequently, aggregate assessments of memory and belief accuracy may depend on how often recollection-based retrieval is engaged at test.

The familiarity backfire and integrative encoding accounts are similar in that they are both based on dual-process models of memory. They both assume that recollection of correct information opposes the familiarity of misinformation. However, these accounts differ in their assumptions about the consequences of retrieving misinformation while studying correct information, which should occur more often after repeated exposure to misinformation. The familiarity backfire account proposes that correction-cued retrievals will increase later familiarity-based source misattributions, especially when recollection is impaired. Conversely, the integrative encoding account proposes that correction-cued retrievals will enable associative encoding of the relationship of information details. This, in turn, promotes subsequent recollection of content and source information, thus counteracting recollection impairments. Under this account, familiarity can still exert an unwanted influence when recollection fails, but recollection should be more likely following integrative encoding. These different assumptions lead to competing predictions about the effects of repeated exposure to fake news headlines on the efficacy of corrections for younger and older adults.

Older adults recollect less well than younger adults but familiarity remains invariant (e.g., Jennings & Jacoby, 1993). From the familiarity backfire perspective, repetition-induced fake news familiarity should lead to more memory errors for older adults because they would be less able to use recollection to oppose familiarity. In contrast, repetitions may increase recollection of fake news for younger adults leading to more rejections. Supporting this prediction, research has shown that increasing repetitions of items from a non-target source increases subsequent false alarm recognition memory errors for older adults and decreases such errors for younger adults (Jacoby, 1999). The integrative encoding perspective also predicts overall poorer memory for older than younger adults because older adults should experience impairments in associative encoding of fake and real news details and subsequent recollection. However, older adults’ subsequent memory accuracy should still benefit from fake news repetitions when they promote detection of corrections that lead to integrative encoding and recollection of the correction episode. Similar patterns of age-related memory differences have been observed in episodic memory updating studies using word pairs (Wahlheim, 2014) and movies of everyday events (Wahlheim & Zacks, 2019) for stimuli.

While the memory literature guides predictions for age-related differences in the effects of repeating fake news before corrections, the literature on how older adults interact with fake news leads to less clear predictions. Most of the latter work has focused on truth discernment (i.e., real/fact or fake/myth) and has shown conflicting evidence about age-related differences. Some studies have shown that older adults are better at discerning real from fake news than younger adults (Allcott & Gentzkow, 2017; Arin et al., 2023; Brashier & Schacter, 2020; Roozenbeek et al., 2020), while other studies found no age differences (Abraham & Mandalaparthy, 2021; Pehlivanoglu et al., 2022). Older adults could be more susceptible to false beliefs and familiarity-based memory errors when source memory fails (Law et al., 1998; but see Mutter et al., 1995; Parks & Toth, 2006). But older adults have also been shown to resist repetition-induced belief errors when faced with information contradicting prior knowledge (Brashier et al., 2017), unlike younger adults (Fazio et al., 2015). In sum, older adults could be more susceptible to false beliefs based on familiarity, but their existing knowledge of world events reported in the news could also protect them.

In studies that correct misinformation, research suggests that correction effects are less durable for older adults. For example, a study examining the mechanisms of belief updating showed that older adults were worse than middle-aged adults at sustaining post-correction beliefs that false claims were inaccurate (Swire et al., 2017). Additionally, older adults have been shown to misremember disproportionately more myths as facts after repeated than single corrections than younger adults (Skurnik et al., 2005). If beliefs are partly based on memory for veracity information learned from corrections, as suggested by recent studies (Kemp et al., 2022b; Swire-Thompson et al., 2023; Wahlheim et al., 2020), then predictions about age differences in memory outcomes from the familiarity backfire and integrative encoding accounts may also extend to the accuracy of beliefs in retrieved details.

### The present study

The primary aim of the present study was to characterize age-related differences in post-correction fake news repetition effects on memory and belief accuracy for retrieved details. To do this, we used a three-phase fake news correction paradigm including headlines from the internet presented in formats similar to news headlines on search engines and social media sites. Fake news headline content had been posted on the internet as real news, and real news details had appeared in corrections on legitimate fact-checking websites. In Phase 1, participants rated their familiarity with and the accuracy of real and fake news headlines of unclear veracity. In Phase 2, participants read real news headlines that affirmed real news and corrected fake news. Participants indicated when they detected that real news headlines corrected fake news headlines from Phase 1. After Phase 2, an approximately 1–3-day retention interval occurred to reduce recollection-based retrieval, thus creating theoretically appropriate conditions for examining familiarity-based memory errors. Finally, in Phase 3, participants completed a recognition (Experiment 1) or cued recall (Experiment 2) test. For both test types, each trial measured 1) retrieval of real news details, 2) accuracy ratings for retrieved details, 3) memory for whether retrieved details corrected fake news, and 4) retrieval of fake news details.

Note that belief accuracy on the Phase 3 test was operationalized as the difference in accuracy ratings for correctly retrieved real news and incorrectly retrieved fake news. Larger differences in accuracy ratings indicated greater belief accuracy. Also, fake news exposure was manipulated by presenting headlines once or thrice in Phase 1. This procedure allowed us to assess post-correction fake news repetition effects on memory and the belief accuracy of retrieved details. It also allowed us to assess conditional retrieval of real news based on whether fake news was also retrieved. Finally, we evaluated how fake news repetitions affected the contributions of recollection and familiarity to retrieval using a hierarchical Bayesian multinomial processing tree (MPT) approach.

Based on findings from related work on fake news corrections (Kemp et al., 2022a, 2022b; Wahlheim et al., 2020), we expected repeated fake news to improve detection of corrections in Phase 2 and subsequent memory for real news to the extent that participants could recollect that fake news was corrected in Phase 3. When detected corrections are not later remembered as such, we expected that repeated fake news would create more interference, and thus more false recognition and intrusions of fake news, consistent with findings from paired-associated learning (Wahlheim, 2014; Wahlheim et al., 2019). As mentioned previously, repeating items from a non-target source can improve memory accuracy for younger adults and impair memory accuracy for older adults (Jacoby, 1999). In that study, participants studied visual then auditory word lists with one to three presentations in the visual list. A recognition exclusion task required participants to reject seen words and endorse heard words. False alarms to seen words decreased with repetitions for younger adults and increased with repetitions for older adults. These findings suggested that older adults were less able to use recollection to reject seen words, and that repetitions increased the familiarity-based errors for those words. Taken with the aforementioned findings from the fake news correction studies, these findings lead to the prediction that fake news repetitions should improve detection of corrections and subsequent memory accuracy. However, when recollection fails in the Phase 3 test, which should occur more for older adults, fake news repetitions should further diminish the accuracy of memory for real and fake news details. Converging evidence for such age-related recollection differences should also emerge in MPT estimates.

Finally, earlier work has shown that participants can reasonably discern retrieved real news details from retrieved fake news details, especially when the type of corrections strongly promoted integrative encoding (e.g., Kemp et al., 2022b; Wahlheim et al., 2020). Such discernment, referred to as belief accuracy, was also greater when detected corrections were later remembered. Based on these prior findings, we expected that participants would rate retrieved real news details as being more accurate than retrieved fake news details. We also expected that the accuracy ratings for retrieved real news details would be even higher when participants could recognize and recall fake news details and remember that those details were corrected earlier. We expected this pattern for both age groups, with the possibility that older adults would show lower belief accuracy (i.e., a smaller difference in accuracy ratings for retrieved real and fake news details) if they were less able than younger adults to recollect the source of headline details in the service of evaluating veracity.

## Experiment 1

Experiment 1 characterized the effects of repeating fake news on the efficacy of corrections for subsequent recognition memory and belief accuracy in younger and older adults. We assessed the contributions of recollection and familiarity-based retrieval and tested predictions from familiarity backfire and integrative encoding accounts.

### Methods

All stimuli, data, and analysis scripts are available here: https://osf.io/vqwtu/ (Kemp et al., 2023). These experiments were approved by the Institutional Review Board at The University of North Carolina at Greensboro (UNCG; IRB-FY21-179). In both experiments, we use specific terminology to refer to the measures in each of the phases. Table 1 provides a glossary of terms.

**Table 1:** Glossary of terms for dependent measures in Phases 2 and 3

Experiment	Phase	Term	Definition
1 & 2	2	Detecting Corrections	Classifying a headline in Phase 2 as a correction of fake news from Phase 1
	3	Remembering Corrections/Correction Remembered	Classifying a headline in Phase 3 as being from a Phase 2 correction of fake news from Phase 1
	3	Correction Not Remembered	Not classifying a headline in Phase 3 as being from a Phase 2 correction of fake news from Phase 1
1	3	Correct Recognition of Real News	Identifying a real news headline from Phase 2 as real news
	3	False Recognition of Fake News	Identifying a fake news headline from Phase 1 as real news
	3	Correct Recognition of Fake News/Fake News Recognized	Identifying a fake news headline from Phase 1 as fake news after remembering a Phase 2 correction
	3	Fake News Not Recognized	Not identifying a fake news headline from Phase 1 as fake news after remembering a Phase 2 correction
2	3	Correct Recall of Real News	Recalling a real news headline detail from Phase 2 as real news
	3	Intrusions of Fake News	Recalling a fake news headline detail from Phase 1 as real news
	3	Correct Recall of Fake News/Fake News Recalled	Recalling a fake news headline detail from Phase 1 as fake news after remembering a Phase 2 correction
	3	Fake News Not Recalled	Not recalling a fake news headline detail from Phase 1 as fake news after remembering a Phase 2 correction

All the terms above pertain to responses made in the conditions with corrections of fake news (see text for details)

### Participants

The stopping rule was to obtain usable data from at least 102 younger and 102 older adults. Given the absence of prior research on age-related differences in post-correction recognition of headlines, we determined the sample size based on available time and financial resources. Notably, the sample size was triple that of typical recruitment for aging studies using the same hierarchical Bayesian MPT models employed in the current work (Bartsch et al., 2019; Loaiza & Srokova, 2020). This sample size also ensured equal administration of three experimental formats across subjects. Participants were recruited online from Prolific (www.prolific.ac) with pre-screening for high approval rating, gender balance, US nationality, US residence at the time of testing, and ages between 18–35 years (younger adults) or 65–75 years (older adults). The advertisement specified that participation required downloading software that was only compatible with desktop or laptop devices. Participants received $10 for completing two sessions.

In total, we tested 135 younger and 124 older adults. The final sample included 102 younger adults (62 women, 40 men) ages 18–29 years (*M* = 22.70, *SD* = 2.92) and 102 older adults (62 women, 40 men) ages 65–75 years (*M* = 69.10, *SD* = 2.76). Data from the remaining 33 younger and 22 older adults were collected but were not included in the analyses for the following reasons: 24 younger and 11 older did not return for the second session, four younger and four older were exposed to the procedure in the first session multiple times by re-opening it before starting the second session, four younger and three older did not complete the first session, one younger and three older completed the study after the target sample size was reached, and one older did not complete the second session.

### Design

This experiment used a mixed factorial design, including Age as a between-subjects variable with younger and older adults as levels. The within-subjects variable was Headline Type with three levels determined by the relationship between headline veracity in Phases 1 and 2. First, an affirmed real news condition included one real news headline in Phase 1 and a repetition of that headline in Phase 2 [Real (1 ×), Real (1 ×)]; a single-exposure fake news correction condition included one fake news headline in Phase 1 and a real news correction of that headline in Phase 2 [Fake (1 ×), Real (1 ×)]; and finally, a repeated-exposure fake news correction condition included three presentations of the same fake news headline in Phase 1 and a real news correction of that headline in Phase 2 [Fake (3 ×), Real (1 ×)].

### Materials and procedure

**Fig. 1:**
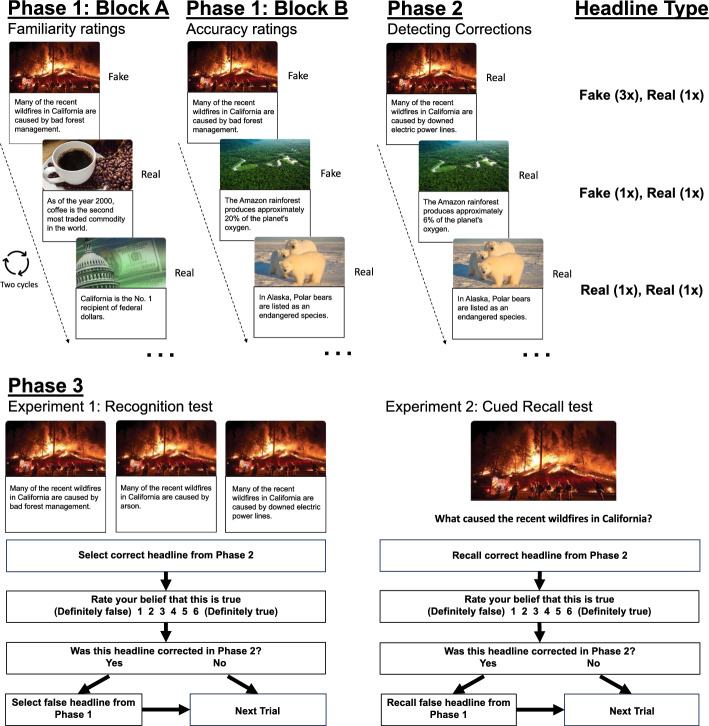
Schematic of the procedure. A schematic overview of the headline types and trial structures. In Phase 1, participants viewed real and fake news headlines of unclear veracity across two seamless blocks. In Block A, participants viewed real and fake news headlines twice and rated each headline’s familiarity. In Block B, the fake news headlines from Block A and new fake and real news headlines appeared once; participants rated each headline’s accuracy. In Phase 2, participants viewed real news headlines of clear veracity that corrected fake news and affirmed real news from Phase 1; Participants attempted to detect corrections of fake news. The Phase 3 trial structures differed between experiments. Experiment 1 included a three-alternative-forced-choice recognition test; on each trial, participants attempted to identify the real news headline from Phase 2, rated the accuracy of their choice, indicated if the recognized headline corrected fake news from Phase 1, and, if so, attempted to identify the fake news headline from Phase 1 from the remaining two headlines. Experiment 2 included a cued recall test; on each trial, participants attempted to recall the real news detail from Phase 2, rated the accuracy of the recalled detail, indicated if it corrected fake news from Phase 1, and if so, attempted to recall the fake news detail from Phase 1

Figure 1 shows example stimuli, experimental conditions, and procedural details. The stimuli comprised 60 headline pairs taken from fact-checking websites (i.e., politifact.com and snopes.com). Each pair featured a real and fake news headline on the same unique topic. Fake news headlines included a false detail, and real news headlines included a true detail that corrected the false detail. All fake news headlines were initially presented by the media as being accurate. The headline format mimicked news updates found on internet search engines and social media sites. Both real and fake news headlines appeared beneath an image related to the topic. Of the 60 pairs, 45 were critical items and 15 were fillers that appeared in the first phase (see below). Critical items were counterbalanced by rotating three sets of 15 pairs through the Headline Type conditions, resulting in three experimental formats. Headlines appeared equally often in each condition across participants.

Stimulus presentation was controlled using Inquisit software (*Inquisit 5*, 2016). Participants completed the experiment on their laptop or desktop computers unsupervised. To ensure device consistency among participants, the program terminated the experiment if it detected that participants were not using a desktop or laptop computer. The experiment included three phases. In each phase, stimuli appeared in a fixed random order with the restriction that no more than three headlines from the same condition appeared consecutively. To control for serial position effects, the average list position for each condition was equated. During interstimulus intervals, a blank screen appeared for 0.5 s, followed by a “Next” button in the center of the screen. To ensure task engagement, participants were required to click that button with a mouse to advance to the next trial.

Fake news exposure in Phase 1 was manipulated by distributing repetitions of fake news headlines across two seamless blocks. Before the first block (Block A), an instruction screen told participants about the upcoming headlines and instructed them to study the headlines for a later test. Block A comprised an equal number of real and fake news headlines. This was achieved by interspersing real news filler items with the first two presentations of fake news headlines from the condition with three fake news exposures. Block A included 30 unique headlines (15 fake news critical items and 15 real news fillers), each appearing once in two cycles. All 30 headlines appeared once in the first cycle before any headline repeated in the second cycle (60 total presentations in Block A). Participants rated their familiarity with each headline on a scale from 1 (Definitely Unfamiliar) to 6 (Definitely Familiar) by clicking on boxes displayed on the screen. Each headline appeared for 8 s.

Before the second block in Phase 1 (Block B), an instruction screen told participants about the upcoming real and fake news headlines and instructed them to study the headlines for a later test. Participants were told that some headlines would be repetitions from Block A, while others would be new. Block B included 60 headlines, equally split between real and fake news. Thirty real news headlines comprised 15 critical items that were eventually affirmed in Phase 2 [Real (1 ×), Real (1 ×)] and 15 repetitions of fillers from Block A. Thirty fake news headlines corresponded with the two fake news exposure conditions. One set included 15 new fake news headlines that appeared only once in Block B of Phase 1 and were eventually corrected in Phase 2 [Fake (1 ×), Real (1 ×)]; the other set included the third presentation of the 15 fake news headlines that appeared twice in Block A. These were also eventually corrected in Phase 2 [Fake (3 ×), Real (1 ×)]. Participants rated headline accuracy on a scale from 1 (Definitely False) to 6 (Definitely True) by clicking on boxes displayed on the screen. Each headline appeared for 8 s.

Before Phase 2, an instruction screen told participants that they would read real news headlines that would either repeat real news or correct fake news from Phase 1. It further instructed participants to indicate whether the headlines corrected fake news. Fake news corrections included details that conflicted with headlines from Phase 1. Phase 2 comprised 45 headlines, with 15 headlines corresponding to each of the Headline Type conditions. To identify headlines that corrected fake news, participants responded “Yes” or “No” by clicking boxes displayed on the screen. Each headline appeared for 8 s. After participants made a response, the question prompt disappeared, but the headline remained on the screen.

After Phase 2, an instruction screen told participants to start the second session after 48 h and no later than 73 h. Due to unique platform features, some participants started slightly earlier than 48 h after completing the first session. This occurred when participants started the second session two days later, as instructed, but earlier than when they started the first session. The average number of hours between sessions did not differ for younger adults (*M* = 51.81, *SD* = 6.23, *Range* = [43.16–71.37]) and older adults (*M* = 52.11, *SD* = 6.66, *Range* = [43.27–72.67]), *t*(202) = 0.34, *p* = 0.73. During this interval, a research assistant checked each data file to ensure that the participants completed all the trials in the first session. Upon verifying this, participants were then manually placed on a custom list that granted them access to the second session. Three notifications were sent through the Prolific messaging portal to remind participants about returning to complete the second session: once the morning before the session became available, once immediately after the session became available, and again the next morning for participants who had not started the study by then.

Before Phase 3, an instruction screen told participants that their task would be to answer questions about their memory for the headlines they read in the first session and the accuracy of retrieved headlines. Phase 3 included a three alternative forced choice (3AFC) recognition memory test with the 45 critical headline topics. On each trial, three headlines on the same topic appeared beneath the image from the earlier phases. The headlines included: real news from Phase 2, fake news that appeared in Phase 1 for the correction conditions, and fake news containing a plausible detail that we generated anew and did not appear in Phase 1. Note that in the condition where real news headlines repeated across Phases 1 and 2 [Real (1 ×), Real (1 ×)], no fake news details had appeared in Phase 1. The fake news details that appeared in that condition were the details that would have appeared in Phase 1 had the headline topic been assigned to a condition that corrected fake news. The inclusion of the second fake news headline with a plausible extra-experimental detail allowed us to precisely examine source confusion between fake news and real news (see Statistical Methods). The screen position of the real news headline was counterbalanced, ensuring equal distribution across trials, and avoiding more than two consecutive appearances in the same position.

On each trial, participants first attempted to select the real news headline from Phase 2. Next, they rated the accuracy of the chosen headline from 1 (Definitely False) to 6 (Definitely True) by clicking response boxes on the screen. Then, participants indicated via key press if real news in Phase 2 had corrected fake news from Phase 1 by responding “Yes” (1) or “No” (0). After responding “yes,” they indicated which of the remaining headlines was fake news from Phase 1 and then advanced to the next trial. After responding “no,” they advanced to the next trial.

### Statistical methods

In both experiments, we performed all statistical tests using R software (R Core Team, 2022). To examine the effects of headline types, we fitted linear and logistic mixed-effects models using functions from the lme4 package (Bates et al., 2015). Based on signal detection theory (SDT; Green & Swets, 1966) we also characterized detection of corrections in Phase 2 and subsequent memory that corrections had been detected in Phase 3 in terms of sensitivity (*d’*) and response bias (*c*). Sensitivity (*d'*) measures participants' ability to distinguish between signals and noise. Response bias (*c*) measures participants’ tendency to report the presence of a signal when the evidence is weak. A conservative bias (higher values of *c*) indicates a higher threshold for reporting that signals are present, leading to fewer false alarms but more misses. Conversely, a liberal bias (lower values of *c*) indicates a lower threshold, resulting in more hits but also more false alarms. In the present study, more conservative biases indicated that participants were less likely to report that topics were corrected, whereas more liberal biases indicated that participants were more likely to report that topics were corrected. Hit and false alarm rates for each participant were used along with the dprime function from the psycho package (Makowski, 2021) to estimate the parameters. Note that we draw our primary conclusions about differences in detecting and remembering corrections based on age and fake news exposure using the sensitivity estimates in Phases 2 and 3, respectively. We did this because sensitivity is independent of response biases that contaminate raw response proportions. However, we still report the raw response proportions for completeness. Doing so characterizes the response rates that led to SDT parameter estimates as well as the differences in observations contributing to the cells in the conditional recognition and recall results. We performed Wald’s *χ*^2^ hypothesis tests using the Anova function of the car package (Fox & Weisberg, 2019). Finally, we performed pairwise comparisons using the Tukey method in the emmeans package (Lenth, 2021), which controlled for multiple comparisons.

All models included Age and Headline Type as fixed effects, with participants and items as random intercept effects. Given the self-paced access to Phase 3, we controlled for the study-test delay in the mixed-effects models of Phase 3 recall performance by including the amount of time between experimental sessions (i.e., retention interval) as a fixed effect. We removed this variable when its inclusion hindered model convergence. The complete model specifications are in the scripts on the OSF. The significance level was *α* = 0.05.

We additionally fit hierarchical Bayesian MPT models using the TreeBUGS package (Heck et al., 2018) to estimate the contributions of recollection and familiarity to the first responses of the Phase 3 test procedure in the conditions that corrected fake news. The models estimate the probability of these latent cognitive parameters based on the frequency of each response type (i.e., correct recognition/recall of real news from Phase 2, false recognition/intrusions of fake news from Phase 1, and false recognition/recall of details that never appeared). Following similar work (Bartsch et al., 2019; Kemp et al., 2022b) based on dual process models of memory (e.g., Jacoby, 1999), we assumed that participants could correctly recognize/recall real news headlines based on recollection (*Pr*). When recollection fails (1 − *Pr*), participants may be familiar with true and false details from earlier phases (*Pf*), leading to equal probabilities of guessing the details from both headline types. Finally, without familiarity (*Pf*), participants may guess with equal probability among the three response types. The parameters of interest are thus *Pr* and *Pf*, whereas the two guessing parameters were fixed to 0.5 to achieve model identifiability.

The MPT models are hierarchical because they estimate parameters for each participant and are Bayesian because they estimate the parameters’ posterior distributions based on uninformative priors and the data using Markov Chain Monte Carlo sampling. Each model was conducted with 4 chains of 100,000 iterations, with 20,000 iterations for adaptation, 2,000 iterations for burn-in, and a thinning factor of 5. The results showed model convergence and adequate fit to the data. This enabled comparison of the posterior distributions to determine if differences in the parameter estimates across conditions were credible (i.e., the 95% credibility intervals of the differences do not overlap with 0).

## Results and discussion

In both experiments, we refer to the dependent measures involving detecting and remembering corrections as well as retrieving real and fake headline details using specific shorthand terminology (see Table 1 for a glossary). We also report analyses that include the complete set of headline stimuli. However, a reviewer raised the possibility that high pre-existing familiarity with some headlines could have muted the strength of the fake news exposure manipulation. To address this, we conducted a complementary set of analyses removing all headlines rated as definitely familiar in Phase 1 (Block A). The patterns of results from every analysis except one were comparable for the full set and subset of data. We note the discrepancy and report the results from the subset analysis in a footnote.

### Familiarity ratings: Phase 1

Table 2 (top rows) shows familiarity ratings for fake news from the Fake (3 ×), Real (1 ×) condition in the two cycles of Phase 1, Block A. We compared these ratings for younger and older adults across cycles using a model with Age and Cycle as fixed effects. The model indicated a significant effect of Age, *χ*^2^(1) = 18.85, *p* < 0.001, showing higher ratings for younger than older adults, and a significant effect of Cycle, *χ*^2^(1) = 58.76, *p* < 0.001, showing that ratings increased from the first to the second cycle. There was also a significant interaction, *χ*^2^(1) = 8.59, *p* < 0.01, showing that the age difference was greater in the first cycle, *z* ratio = 4.89, *p* < 0.001, than in the second cycle, *z* ratio = 3.61, *p* < 0.001.

**Table 2:** Familiarity ratings for fake news in Phase 1, Block A

Experiment	Age	Cycle 1	Cycle 2
1	Younger	3.17 [2.94, 3.39]	3.28 [3.05, 3.51]
	Older	2.62 [3.29, 2.85]	2.88 [2.65, 3.11]
2	Younger	2.81 [2.59, 3.04]	3.08 [2.86, 3.31]
	Older	2.67 [2.45, 2.90]	2.85 [2.59, 3.04]

95% confidence intervals are displayed in brackets

### Accuracy ratings: Phase 1

Table 3 (top rows) shows accuracy ratings for real and fake news headlines in Phase 1, Block B. A model with Age and Headline Type and as fixed effects indicated significant effects of Age, *χ*^2^(1) = 48.72, *p* < 0.001, and Headline Type,* χ*^2^(2) = 82.39, *p* < 0.001, and no significant interaction *χ*^2^(2) = 4.80, *p* = 0.09. Younger adults made higher overall ratings across all headline types than older adults. Both groups made higher ratings for real than fake news, smallest *z* ratio = 4.77, *p* < 0.001, and for fake news that appeared thrice compared to once, *z* ratio = 4.31, *p* < 0.001. These results suggest that younger adults were less skeptical when evaluating the accuracy of headlines, both groups could generally discern real from fake news details, and repeating fake news created an illusion that it was more accurate (Hasher et al., 1977; Hassan & Barber, 2021).

**Table 3:** Accuracy ratings for real and fake news in Phase 1, Block B

		Headline type		
Experiment	Age	Real (1 ×)	Fake (1 ×)	Fake (3 ×)
1	Younger	3.81 [3.64, 3.97]	3.54 [3.37, 3.71]	3.72 [3.55, 3.89]
	Older	3.47 [3.30, 3.63]	3.11 [2.94, 3.28]	3.23 [3.06, 3.39]
2	Younger	3.69 [3.52, 3.85]	3.39 [3.23, 3.56]	3.51 [3.34, 3.67]
	Older	3.50 [3.34, 3.66]	3.23 [3.07, 3.40]	3.31 [3.14, 3.47]

95% confidence intervals are displayed in brackets

### Correction classifications: Phases 2 and 3

**Fig. 2:**
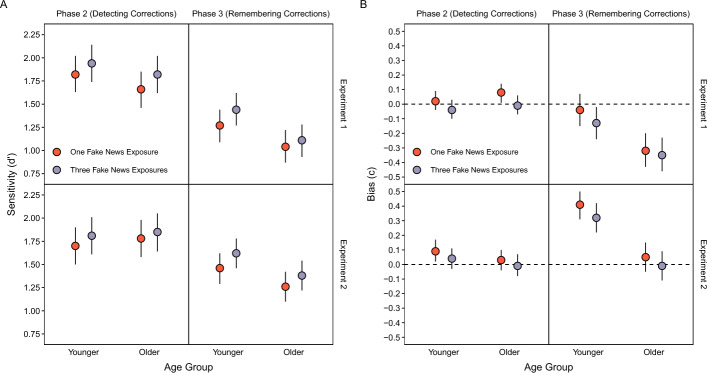
Signal detection parameter estimates for correction classifications in Phases 2 and 3. Sensitivity (**A**) and bias (**B**) estimates derived from mixed effects models with 95% confidence intervals (error bars). Bias estimates (**B**) are conservative when they are above zero and liberal when they are below zero

Table 4 shows correction classifications that participants made to indicate when they detected corrections of fake news in Phase 2 and remembered that fake news was corrected in Phase 3. We computed probabilities of “yes” responses for correction classifications in Phases 2 and 3 across Headline Type conditions (Table 4, top section of rows). Note that these responses are incorrect (false alarms) for the condition affirming real news and correct (hits) for the conditions correcting fake news. Considering both hits and false alarms also allowed us to use signal detection analyses to assess participants’ sensitivity to differences between real and fake news (Fig. 2A, top panels) as well as participants’ response bias (Fig. 2B, top panels). We calculated parameter estimates for both types of fake news corrections by treating “yes” responses in each of those conditions as separate hit rates.

**Table 4:** Detecting corrections in Phase 2 and remembering corrections in Phase 3

			Headline type		
Experiment	Phase	Age	Real (1 ×), Real (1 ×)	Fake (1 ×), Real (1 ×)	Fake (3 ×), Real (1 ×)
1	2	Younger	.17 [.14, .21]	.83 [.79, .86]	.85 [.81, .88]
		Older	.18 [.15, .21]	.78 [.74, .82]	.83 [.80, .86]
	3	Younger	.26 [.22, .31]	.77 [.72, .81]	.82 [.78, .85]
		Older	.40 [.34, .46]	.82 [.77, .85]	.84 [.80, .87]
2	2	Younger	.18 [.15, .21]	.79 [.76, .83]	.81 [.77, .84]
		Older	.18 [.15, .22]	.83 [.79, .86]	.84 [.81, .87]
	3	Younger	.09 [.07, .12]	.64 [.58, .70]	.69 [.63, .75]
		Older	.23 [.18, .28]	.74 [.68, .79]	.78 [.73, .82]

The values above are proportions of “Yes” responses for correction classifications in Phases 2 and 3. These responses are accurate for the Fake (1 ×), Real (1 ×) and Fake (3 ×), Real (1 ×) conditions and inaccurate for the Real (1 ×), Real (1 ×) condition. 95% confidence intervals are displayed in brackets

#### Phase 2 (detecting corrections)

We compared detection of corrections in Phase 2 (Table 4, top rows) using a model with Age and Headline Type as fixed effects. The model indicated no significant effect of Age, *χ*^2^(1) = 1.57, *p* = 0.21, a significant effect of Headline Type, *χ*^2^(2) = 2435.34, *p* < 0.001, and a significant interaction, *χ*^2^(2) = 6.11, *p* < 0.05. The probabilities were significantly higher in the conditions with corrected fake news than the condition with affirmed real news, smallest *z* ratio = 42.63, *p* < 0.001. This showed that participants discriminated corrections of fake news from affirmations of real news. The interaction showed that corrections were detected significantly more often when fake news appeared thrice than once for older adults,* z* ratio = 3.39, *p* < 0.01, but such detections did not differ for younger adults, *z* ratio = 1.52, *p* = 0.28.

We further characterized detection responses by comparing signal detection parameter estimates between fake news correction conditions. A model with Age and Headline Type as fixed effects for *d’* (Fig. 2A, top left panel) indicated no significant effect of Age, *χ*^2^(1) = 1.06, *p* = 0.30, a significant effect of Headline Type, *χ*^2^(1) = 16.49, *p* < 0.001, and no significant interaction, *χ*^2^(1) = 0.61, *p* = 0.43. Participants were more sensitive to corrections of fake news that appeared thrice than once. The same model for *c* (Fig. 2B, top left panel) indicated no significant effect of Age, *χ*^2^(1) = 0.87, *p* = 0.35, a significant effect of Headline Type, *χ*^2^(1) = 16.55, *p* < 0.001, and no significant interaction, *χ*^2^(1) = 0.62, *p* = 0.43, showing that participants adopted a more conservative response bias for corrections of fake news that appeared once than thrice. Collectively, these results show that more fake news exposure improved detection of corrections, which did not differ between younger and older adults.

#### Phase 3 (remembering corrections)

We also compared remembering corrections in Phase 3 (Table 4, second rows) using a model with Age and Headline Type as fixed effects. The model indicated significant effects of Age, *χ*^2^(1) = 6.90, *p* < 0.01, and Headline Type, *χ*^2^(2) = 1486.74, *p* < 0.001, and a significant interaction, *χ*^2^(2) = 14.49, *p* < 0.001. The probabilities were higher for older than younger adults. The probabilities were also significantly higher for the conditions with real news that corrected fake news than the condition with affirmed real news, smallest *z* ratio = 20.86, *p* < 0.001, showing that participants discriminated topics for which fake news was corrected from topics for which real news was affirmed. The interaction showed significantly higher probabilities for fake news headlines that appeared thrice than once for younger adults, *z* ratio = 3.26, *p* < 0.01, and no difference between headline conditions for older adults, *z* ratio = 1.90, *p* = 0.14. The interaction also showed no significant age differences within both conditions that corrected fake news, largest *z* ratio = 1.70, *p* = 0.09, and a significantly greater probability for affirmed real news for older than younger adults, *z* ratio = 3.89, *p* < 0.001.

We further characterized remembering corrections by comparing signal detection parameter estimates between fake news correction conditions. The model for *d’* (Fig. 2A, top right panel) indicated significant effects of Age, *χ*^2^(1) = 5.37, *p* = 0.02, and Headline Type, *χ*^2^(1) = 12.06, *p* < 0.001, and no significant interaction, *χ*^2^(1) = 2.71, *p* = 0.10. Memory for topics being associated with corrections, assessed using *d’*, was more accurate for younger than older adults and for corrections of fake news that appeared thrice than one. The same model for *c* (Fig. 2B, top right panel) indicated significant effects of Age, *χ*^2^(1) = 9.90, *p* < 0.01, and Headline Type, *χ*^2^(1) = 12.05, *p* < 0.001, and no significant interaction, *χ*^2^(1) = 2.73, *p* = 0.10. Response bias was more conservative for younger than older adults and for corrections of fake news that appeared once than thrice. Collectively, these results show that more fake news exposure led to more accurate remembering that it was corrected. Such remembering was more precise for younger adults who also showed more conservative reporting of topics being corrected (for another example of such age differences in response bias using a recognition paradigm, see Fraundorf et al., 2019).

### Overall 3AFC recognition memory: Phase 3

We examined the effects of fake news exposure prior to corrections on subsequent memory accuracy by assessing recognition memory for real and fake news details in Phase 3. We assessed memory accuracy by comparing correct recognition of real news headlines as well as false and correct recognition of fake news headlines. We used separate models with Age and Headline Type as fixed effects for each memory measure.

#### Correct recognition of real news

**Fig. 3:**
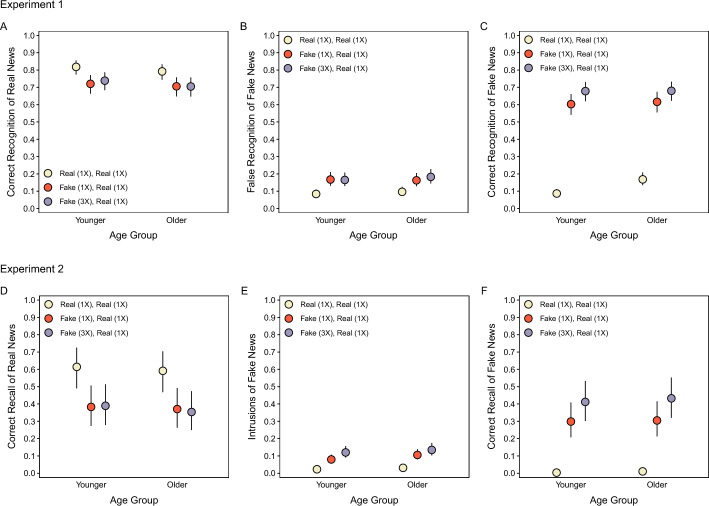
Overall test response probabilities. The top row shows the Experiment 1 probabilities of correct recognition of real news (**A**), false recognition of fake news (**B**), and correct recognition of fake news (**C**). The bottom row shows the Experiment 2 probabilities of correct recall of real news (**D**), intrusions of fake news (**E**), and correct recall of fake news (**F**). Points are estimates derived from mixed effects models with 95% confidence intervals (error bars). Error bars are not visible when they are smaller than point diameters. Note that all responses regarding fake news headlines in the condition that only featured real news in Phases 1 and 2 (panels **B**, **C**, **E**, **F**) are instances when participants “recognized” or “recalled” fake news that did not appear in Phase 1 (see text for details)

Figure 3A displays correct recognition of real news, which refers to when participants chose the real news headline from the three alternatives. The model indicated no significant effect of Age, *χ*^2^(1) = 0.95, *p* = 0.33, a significant effect of Headline Type, *χ*^2^(2) = 77.80, *p* < 0.001, and no significant interaction, *χ*^2^(2) = 0.84, *p* = 0.66. Recognition accuracy was significantly higher for affirmed real news than real news corrections of fake news, smallest *z* ratio = 7.37, *p* < 0.001, and did not differ between correction conditions, *z* ratio = 0.73, *p* = 0.75. These results show that recognition was better when only real news had appeared than when fake news details competed with real news details. Moreover, the lack of a fake news exposure effect suggests that fake news repetitions created offsetting improvements and impairments that depended on detecting and remembering corrections. We address this further on.

#### False recognition of fake news

Figure 3B displays false recognition of fake news, which refers to when participants chose the fake news headline from the three alternatives. For both correction conditions, false recognition of fake news details from Phase 1 reflects errors where veracity information was not retrieved. For the condition with affirmed real news, such errors are instances when participants recognized fake news that did not appear in Phase 1. The model indicated no significant effect of Age, *χ*^2^(1) = 0.29, *p* = 0.59, a significant effect of Headline Type, *χ*^2^(2) = 118.09, *p* < 0.001, and no significant interaction, *χ*^2^(2) = 1.96, *p* = 0.38. False recognition was significantly higher in both correction conditions than the affirmation condition, smallest *z* ratio = 9.25, *p* < 0.001, and did not differ between correction conditions, *z* ratio = 0.90, *p* = 0.64. As for correct recognition, repeating fake news may not have increased false recognition in the aggregate because it led to offsetting improvements and impairments.

#### Correct recognition of fake news

Figure 3C displays correct recognition of fake news, which refers to when participants indicated that fake news was corrected and selected the fake news headline from Phase 1 from the remaining two alternatives. For the affirmed real news condition, this reflects when participants correctly “recognized” fake news headlines without seeing them in Phase 1. The model indicated no significant effect of Age, *χ*^2^(1) = 2.07, *p* = 0.15, a significant effect of Headline Type, *χ*^2^(2) = 1607.29, *p* < 0.001, and a significant interaction, *χ*^2^(2) = 33.98, *p* < 0.001. Both age groups recognized fake news significantly better when it appeared thrice than once, smallest *z* ratio = 3.35, *p* < 0.01, and when it was corrected than when it did not appear, smallest *z* ratio = 22.88, *p* < 0.001. The interaction indicated that older adults “recognized” fake news that did not appear in Phase 1 more than younger adults, *z* ratio = 4.46, *p* < 0.001, which may reflect their greater knowledge of (and hence familiarity with) news content than younger adults (Brashier et al., 2017).

### Recognition in Phase 3 for corrections detected in Phase 2 conditionalized on fake news recognition or remembering corrections in Phase 3

We conducted conditional analyses of correct and false recognition to determine if repeating fake news led to offsetting improvements and impairments that depended on detection of and memory for corrections. We are primarily interested in how fake news accessibility during corrections is associated with subsequent memory accuracy. We thus focused the following analyses on instances where corrections were detected in Phase 2 in the corrected fake news conditions. We assumed that correction detection was often based on retrieval of fake news and that those instances promoted associative encoding of fake and real news details, based on our prior work (Kemp et al., 2022b). We expected that detecting corrections in Phase 2 would enhance memory for real news when fake news could be later recognized and impair memory for real news when fake news could not be later recognized. We assumed that recognizing fake news in Phase 3 reflected recollection of corrections because participants had to respond “yes” that a topic was corrected to receive the opportunity to recognize fake news as such. We do not report conditional analyses for the cells where corrections were not detected in Phase 2 because the data were too sparse to interpret.

#### Correct recognition of real news

**Fig. 4:**
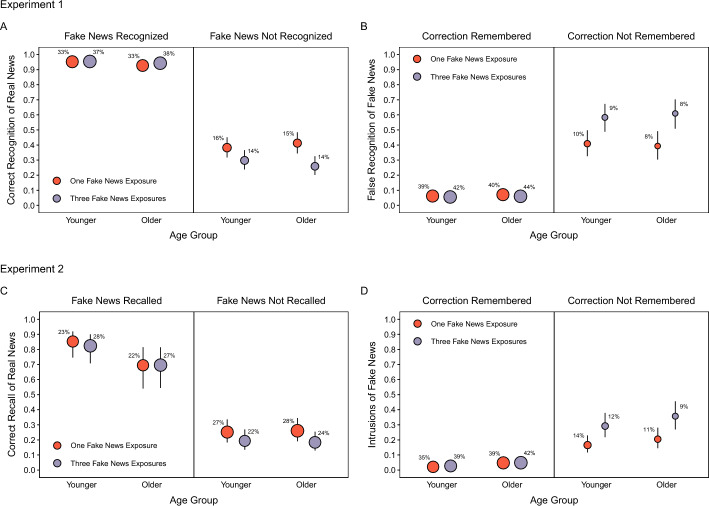
Test response probabilities for detected corrections conditionalized on retrieval of fake news and remembering corrections*.* The top row shows the Experiment 1 probabilities of correct recognition of real news conditionalized on fake news recognition (**A**) and false recognition of fake news conditionalized on remembering corrections (**B**). The bottom row shows the Experiment 2 probabilities of correct recall of real news conditionalized on fake news recall (**C**) and intrusions of fake news conditionalized on remembering corrections (**D**). Points are estimates derived from mixed effects models with 95% confidence intervals (error bars). Error bars are not visible when they are smaller than point diameters. The point areas and corresponding percentages indicate the observations contributing to each cell. Percentages that do not sum to 100% within age group reflect rounding error

We examined whether correct recognition of real news depended on correct recognition of fake news by conditionalizing real news recognition on fake news recognition when participants indicated remembering a correction (Fig. 4A). We used a model including Age, Fake News Recognition, and Headline Type as fixed effects. Fake news recognition had two levels: correct recognition hits (fake news recognized; left panel) and incorrect recognition misses (fake news not recognized; right panel).

Table 5 shows the model results. A significant effect of Fake News Recognition, *χ*^2^(1) = 991.37, *p* < 0.001, showed that real news recognition was higher when fake news was recognized than when it was not. Also, a significant Fake News Recognition × Headline Type interaction, *χ*^2^(1) = 15.54, *p* < 0.001, showed that when fake news was recognized, there was no difference between the headline types, *z* ratio = 1.04, *p* = 0.30; conversely, when fake news was not recognized, real news recognition was significantly lower when fake news appeared thrice than once, *z* ratio = 4.54, *p* < 0.001. Taken with the previous fake news recognition rates, these results show that more fake news exposure led to real news recognition receiving the benefits associated with fake news recognition on more trials (left panel, larger point sizes). However, this improvement was offset by the larger magnitude of impairment after additional fake news exposures when fake news was not recognized (right panel, lower point heights).

**Table 5:** Model results for correct recognition of real news in Phase 3 for detected corrections in Phase 2 conditionalized on fake news recognition in Phase 3: experiment 1

Effect	*χ*^2^	*df*	*p*
Age	3.20	1	= .07
Fake News Recognition	991.37	1	< .001
Headline Type	5.73	1	= .02
Age × Fake News Recognition	3.24	1	= .07
Age × Headline Type	0.27	1	= .60
Fake News Recognition × Headline Type	15.54	1	< .001
Age × Fake News Recognition × Headline Type	2.74	1	= .10

#### False recognition of fake news

We next examined the dependence between false recognition of fake news and memory for corrections by conditionalizing false recognition of fake news on remembering corrections (Fig. 4B). We used the same modeling approach as in the previous analyses. The two levels of remembering corrections were correct and incorrect classifications of corrected topics (Correction Remembered and Correction Not Remembered, respectively). We could not conditionalize these responses on fake news recognition accuracy, as in the conditional analyses of correct real news recognition, because the fake news headlines from Phase 1 did not appear as response options after being falsely recognized as real news.

Table 6 shows the model results. A significant effect of Remembering Corrections, *χ*^2^(1) = 436.25, *p* < 0.001, showed lower false recognition of fake news when corrections were remembered than when they were not. There was also a significant Remembering Corrections × Headline Type interaction, *χ*^2^(1) = 20.91, *p* < 0.001. When corrections were remembered, false recognition did not differ between the headline types, *z* ratio = 1.06, *p* = 0.29; conversely, when corrections were not remembered, false recognition was significantly higher for fake news that appeared thrice than once, *z* ratio = 4.95, *p* < 0.001. These patterns mirror those for correct recognition of real news in showing that more fake news exposure led to more trials with fewer errors when corrections were remembered (left panel, larger point sizes), but also led to larger magnitudes of errors when corrections were not remembered (right panel, higher point heights).

**Table 6:** Model results for false recognition of fake news in Phase 3 for detected corrections in Phase 2 conditionalized on remembering corrections in Phase 3: experiment 1

Effect	*χ*^2^	*df*	*p*
Age	0.17	1	= .68
Remembering Corrections	436.25	1	< .001
Headline Type	4.04	1	= .05
Age × Remembering Corrections	0.09	1	= .76
Age × Headline Type	0.14	1	= .71
Remembering Corrections × Headline Type	20.91	1	< .001
Age × Remembering Corrections × Headline Type	0.65	1	= .42

### Recollection and familiarity process estimates for Phase 3 recognition

We used the MPT model described previously to assess potential differences in the contributions of recollection and familiarity to headline recognition. Table 7 (top section) shows the parameter estimates for recollection and familiarity from the MPT model. For recollection, there were no credible effects of Age or Headline Type. For familiarity, older adults showed credibly lower familiarity than younger adults for fake news presented once (0.10 [0.01, 0.20]), and credibly higher familiarity for fake news that appeared thrice than once (0.10 [0.01, 0.19)]. The age difference suggests that older adults experienced increased familiarity with fake news following repeated exposure.

**Table 7:** Posterior parameters and differences estimated from MPT models

			Headline Type		
Experiment	Parameter	Age	Fake (1 ×), Real (1 ×)	Fake (3 ×), Real (1 ×)	*Repetition effect*
1	Recollection	Younger	0.47 [0.39, 0.55]	0.49 [0.39, 0.58]	0.02 [− 0.07, 0.10]
		Older	0.46 [0.36, 0.55]	0.44 [0.36, 0.52]	− 0.02 [− 0.10, 0.07]
		*Age effect*	0.01 [− 0.11, 0.13]	0.04 [− 0.08, 0.17]	
	Familiarity	Younger	0.70 [0.64, 0.76]	0.73 [0.67, 0.79]	0.03 [− 0.06, 0.11]
		Older	0.61 [0.53, 0.67]	0.70 [0.64, 0.75]	**0.10 [0.01, 0.19]**
		*Age effect*	**0.10 [0.01, 0.20]**	0.03 [− 0.05, 0.11]	
2	Recollection	Younger	0.31 [0.25, 0.37]	0.26 [0.19, 0.33]	− 0.05 [− 0.12, 0.02]
		Older	0.27 [0.21, 0.32]	0.22 [0.16, 0.28]	− 0.05 [− 0.11, 0.02]
		*Age effect*	0.04 [− 0.04, 0.12]	0.04 [− 0.05, 0.13]	
	Familiarity	Younger	0.02 [0.00, 0.07]	0.16 [0.07, 0.23]	**0.14 [0.05, 0.22]**
		Older	0.06 [0.01, 0.14]	0.09 [0.03, 0.16]	0.03 [− 0.07, 0.12]
		*Age effect*	− 0.04 [0.12, 0.03]	0.07 [− 0.04, 0.17]	

Effects refer to differences between age groups and fake news repetitions. Effects in bold are credible (i.e., their 95% credibility intervals do not overlap with 0). 95% credibility intervals are displayed in brackets

### Accuracy ratings for recognized headlines: Phase 3

**Fig. 5:**
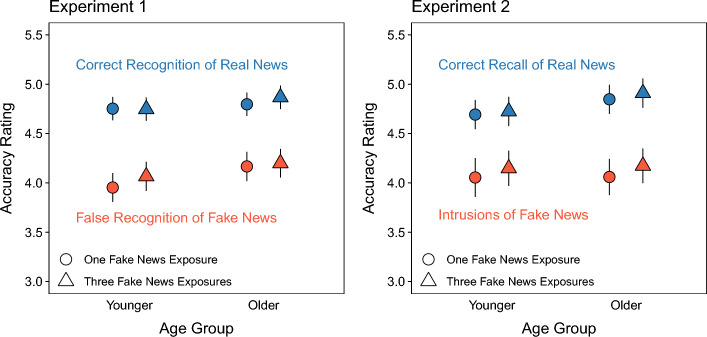
Accuracy ratings for retrieved headline details in Phase 3. Accuracy rating estimates derived from mixed effects models with 95% confidence intervals (error bars) in Experiment 1 (left panel) and Experiment 2 (right panel)

We next examined how fake news exposure affected belief accuracy for fake news correction headline types that participants recognized as real news in Phase 3. To assess belief accuracy, we compared accuracy ratings for correct recognition of real news from Phase 2 and false recognition of fake news from Phase 1 (Fig. 5, left panel).^1^ As described earlier, belief accuracy was indicated by the extent to which accuracy ratings were higher for correct recognition and lower for false recognition; larger differences indicated greater belief accuracy. A model with Age, Response Type, and Headline Type as fixed effects indicated a significant effect of Response Type, *χ*^2^(1) = 462.75, *p* < 0.001, showing higher accuracy ratings for correctly recognized real news than falsely recognized fake news. Table 8 shows that no other effects were significant. The absence of any other significant effects indicated that belief accuracy did not differ based on age group or fake news exposure.

**Table 8:** Model results for accuracy ratings in Phase 3: experiment 1

Effect	*χ*^2^	*df*	*p*
Age	2.32	1	= .13
Headline Type	2.85	1	= .09
Response Type	462.75	1	< .001
Age × Headline Type	0.61	1	= .44
Age × Response Type	2.02	1	= .15
Headline Type × Response Type	0.44	1	= .51
Age × Headline Type × Response Type	1.77	1	= .18

**Fig. 6:**
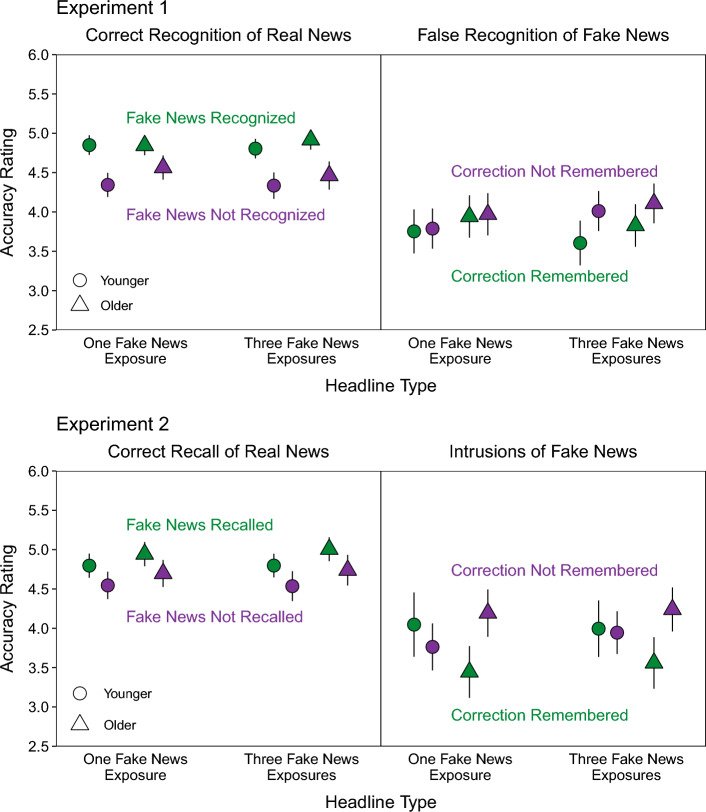
Accuracy ratings for retrieved details conditioned on real news retrieval and remembering corrections. Accuracy rating estimates derived from mixed effects models with 95% confidence intervals (error bars) in Experiment 1 (top panels) and Experiment 2 (bottom panels)

We further assessed belief accuracy by conducting conditional analyses of accuracy ratings (Fig. 6, top panels). Accuracy ratings for correct recognition of real news were conditionalized on recognition of fake news (left panel). The model included Age, Headline Type, and Fake News Recognition as fixed effects. A significant effect of Fake News Recognition, *χ*^2^(1) = 138.36, *p* < 0.001, showed higher accuracy ratings when fake news was recognized than when it was not. Also, a significant Age × Fake News Recognition interaction, *χ*^2^(1) = 4.32, *p* = 0.04, showed that the age difference was nominally smallest after one fake news exposure. Table 9 (top section) shows that no other effects were significant.

**Table 9:** Model results for conditionalized accuracy ratings for correction recognition of real news and false recognition of fake news in Phase 3: experiment 1

Response type	Effect	*χ*^2^	*df*	*p*
Correct recognition of real news	Age	1.41	1	= .24
	Headline Type	< .01	1	= .92
	Fake News Recognition	138.36	1	< .001
	Age × Headline Type	3.26	1	= .07
	Age × Fake News Recognition	4.32	1	= .04
	Headline Type × Fake News Recognition	0.93	1	= .33
	Age × Headline Type × Fake News Recognition	2.66	1	= .10
False recognition of fake news	Age	2.58	1	= .11
	Headline Type	0.48	1	= .49
	Remembering Corrections	6.77	1	< .001
	Age × Headline Type	0.07	1	= .80
	Age × Remembering Corrections	0.23	1	= .63
	Headline Type × Remembering Corrections	5.42	1	= .02
	Age × Headline Type × Remembering Corrections	0.19	1	= .66

Finally, accuracy ratings for false recognition of fake news were conditionalized on remembering corrections (right panel). The model included Age, Headline Type, and Remembering Corrections as fixed effects. There was a significant effect of Remembering Corrections, *χ*^2^(1) = 6.77, *p* < 0.001, and a significant Headline Type × Remembering Corrections interaction, *χ*^2^(1) = 5.42, *p* = 0.02. When fake news appeared once, accuracy ratings were not significantly different based on remembering corrections, *t*(1250) = 0.32, *p* = 0.75. However, when fake news appeared thrice, accuracy ratings were significantly lower when corrections were remembered than when they were not, *t*(1258) = 3.50, *p* < 0.001. Table 9 (bottom section) shows that no other effects were significant.

## Discussion

Experiment 1 showed that the lack of fake news exposure effects on overall correct recognition of real news and false recognition of fake news (Fig. 3A, B) reflected offsetting improvements and impairments that depended on recognition of fake news and remembering corrections (Fig. 4A, B). Repeating fake news improved sensitivity to detecting corrections in Phase 2 (Fig. 2A), remembering corrections in Phase 3 (Fig. 2A), and recognition of fake news in Phase 3 (Fig. 3C). These memory outcomes were associated with comparable benefits to accurate recognition of headlines, comprising increased correct recognition of real news and decreased false recognition of fake news (Fig. 4A, B). However, when fake news was not recognized and corrections were not remembered in Phase 3, more fake news exposure impaired recognition accuracy (Fig. 4A, B). These effects did not differ between age groups. However, younger adults remembered corrections more precisely and classified headline topics as corrected more conservatively (Fig. 2A, B).

The findings from Experiment 1 are consistent with work showing that detecting corrections was associated with improved real news recall when fake news was also recalled and impaired real news recall when fake news details were not recalled (Kemp et al., 2022b). These findings are also compatible with the integrative encoding view (Ecker et al., 2017; Wahlheim et al., 2020), which assumes that familiarity-based memory errors arise when integrated memories of real and fake news are not recollected (cf. Skurnik et al., 2007). Results from the MPT analyses showed that recollection estimates did not differ between age groups, but fake news repetition increased familiarity estimates for older but not younger adults, suggesting a stronger influence of fake news familiarity on memory for older adults (cf. Jacoby, 1999). However, the consequences of this age difference were unclear given the relatively similar performance on most memory measures for younger and older adults.

Finally, belief accuracy in Phase 3, indicated by higher accuracy ratings for correct than false recognition, did not differ across fake news exposures and age groups (Fig. 5, left panel). Recognizing corrected fake news was associated with greater belief accuracy in the form of higher accuracy ratings for recognized real news (Fig. 6, top left panel). This benefit was slightly greater for younger than older adults. Remembering that fake news was corrected was associated with greater belief accuracy in the form of lower accuracy ratings for false recognition of fake news, but only for fake news that appeared thrice in Phase 1 (Fig. 6, top right panel). Collectively, these results suggest that younger and older adults used memory for headline veracity inferred from correction detection in Phase 2 as a basis for their beliefs in retrieved details. Their use of this mnemonic basis varied based on fake news exposure, but the exact mechanism driving such interactions awaits further investigation.

## Experiment 2

Experiment 1 provided an initial characterization of the effects of correcting repeated fake news on memory and belief accuracy in younger and older adults. We began our investigation using a recognition memory task because prior work showed that a similar task was sensitive to age-related recollection differences (Jacoby, 1999). However, recognition accuracy for real and fake news was highly similar for both age groups. This was surprising because although age-related memory differences are smaller in tasks with environmental support (Fraundorf et al., 2019), our task required source monitoring that would typically be impaired in older adults (Danckert & Craik, 2013; but see Rhodes et al., 2019).

One possibility is that our online study attracted highly motivated and capable older adults (Ryan & Campbell, 2021). Another possibility is that the task was sufficiently supportive and that older adults would have shown poorer memory if the task placed higher requirements on recollection-based retrieval. We examined the latter possibility here by recruiting participants from the same online platform and replacing the recognition test with a cued recall test. Even if older adults who participate online are more motivated and capable, a final cued recall test that requires self-generated retrieval and source monitoring may still be sensitive to age-related recollection differences. Finally, we expected to observe conditional recall patterns similar to prior findings, with any age-related deficits reflecting poorer recollection of corrections for older than younger adults (cf. Wahlheim, 2014; Wahlheim & Zacks, 2019).

### Participants

The stopping rule was to collect usable data from 102 younger and 102 older adults for the same reasons as in Experiment 1. New participants were recruited and pre-screened as in Experiment 1. In total, we tested 131 younger and 150 older adults. The final sample included 102 younger adults (41 women, 56 men, 5 gender diverse) ages 18–34 years (*M* = 23.60, *SD* = 2.96) and 102 older adults (56 women, 46 men) ages 62–75 years (*M* = 68.17, *SD* = 3.01). Data from the remaining 29 younger and 48 older adults were excluded for the following reasons: 15 younger and 12 older did not return to the second session, nine younger and 22 older did not complete the first session, five younger and 13 older did not complete the second session, and one older adult was exposed to the procedure in the first session multiple times.

### Design, materials, and procedure

The design, materials, and procedure shared many features with Experiment 1, but there were two key differences. First, participants were instructed to start the second session 24 h after completing the first session and could start up to 49 h later. We reduced the retention interval from Experiment 1 to prevent floor performance on the final test. The average number of hours between sessions was not significantly different for younger adults (*M* = 26.89, *SD* = 4.73, *range* = [23.53–47.25]) and older adults (*M* = 27.29, *SD* = 5.98, *range* = [22.95–48.80]), *t*(202) = 0.34, *p* = 0.73. Second, Phase 3 included a cued recall test. Each test item showed the picture from the headline above a prompt to recall the missing real news detail from Phase 2 (Fig. 1). After typing a response, participants rated the accuracy of the detail they recalled on a scale from 1 (Definitely False) to 6 (Definitely True) by clicking a box on the screen. Next, they indicated whether real news in Phase 2 corrected fake news from Phase 1 by responding “Yes” (1) or “No” (0) via key press. After responding “yes,” they attempted to recall the fake news detail from Phase 1 by typing a response and then they advanced to the next trial. After responding “no,” they advanced to the next trial.

## Results and discussion

### Familiarity ratings: Phase 1

Table 2 (bottom rows) shows familiarity ratings for fake news from the Fake (3 ×), Real (1 ×) condition in the two cycles of Phase 1, Block A. We compared these ratings for younger and older adults across cycles using a model with Age and Cycle as fixed effects. The model indicated no significant effect of Age, *χ*^2^(1) = 2.88, *p* = 0.09, and a significant effect of Cycle, *χ*^2^(1) = 83.65, *p* < 0.001, showing that ratings increased from the first to second cycle. A significant interaction, *χ*^2^(1) = 4.00, *p* < 0.05, showed that ratings did not differ between age groups on the first presentation, *z* ratio = 1.21, *p* = 0.23, but were significantly greater for younger adults on the second presentation, *z* ratio = 2.08, *p* = 0.04.

### Accuracy ratings: Phase 1

Table 3 (bottom rows) shows accuracy ratings for real and fake news headlines in Phase 1, Block B. A model with Age and Headline Type as fixed effects indicated significant effects of Age, *χ*^2^(1) = 9.72, p < 0.01, and Headline Type, *χ*^2^(2) = 67.60, *p* < 0.001, and no significant interaction *χ*^2^(2) = 0.36, *p* = 0.84. Younger adults made higher accuracy ratings than older adults. Both groups made higher ratings for real than fake news, smallest *z* ratio = 5.39, *p* < 0.001, and for fake news that appeared thrice compared to once, *z* ratio = 2.68, *p* = 0.02. These results replicate Experiment 1 and again suggest that younger adults were less skeptical of fake news, both groups could generally discern real from fake news details, and repeating fake news created an illusion that it was more accurate (Hasher et al., 1977; Hassan & Barber, 2021).

### Correction classifications: Phases 2 and 3

#### Phase 2 (detecting corrections)

We compared detection of corrections in Phase 2 (Table 4, bottom section, top rows) using a model with Age and Headline Type as fixed effects. The model indicated no significant effect of Age, *χ*^2^(1) = 3.31, *p* = 0.07, a significant effect of Headline Type, *χ*^2^(2) = 2373.07, *p* < 0.001, and no significant interaction, *χ*^2^(2) = 2.85, *p* = 0.24. The probabilities were significantly higher in the conditions with corrected fake news than the condition with affirmed real news, smallest *z* ratio = 42.63, *p* < 0.001, and did not differ between the conditions with corrected fake news, *z* ratio = 1.61, *p* = 0.24. This shows that participants again discriminated corrections of fake news from affirmations of real news.

We further characterized detection of corrections by comparing signal detection parameter estimates between fake news correction conditions. A model with Age and Headline Type as fixed effects for *d’* (Fig. 2A, bottom left panel) indicated no significant effect of Age, *χ*^2^(1) = 0.17, *p* = 0.68, a significant effect of Headline Type, *χ*^2^(1) = 5.60, *p* = 0.02, and no significant interaction, *χ*^2^(1) = 0.35, *p* = 0.56. Participants were again more sensitive to corrections of fake news that appeared thrice than once. The same model for *c* (Fig. 2B, bottom left panel) indicated no significant effect of Age, *χ*^2^(1) = 1.39, *p* = 0.24, a significant effect of Headline Type, *χ*^2^(1) = 5.67, *p* = 0.02, and no significant interaction, *χ*^2^(1) = 0.35, *p* = 0.55, showing that participants again adopted a more conservative response bias for corrections of fake news that appeared once than thrice. Collectively, these results replicate Experiment 1 in showing that more fake news exposure improved detection of corrections, which did not differ between younger and older adults.

#### Phase 3 (remembering corrections)

We also compared remembering corrections in Phase 3 (Table 4, bottom rows) using a model with Age and Headline Type as fixed effects. The model indicated significant effects of Age, *χ*^2^(1) = 17.42, *p* < 0.001, and Headline Type, *χ*^2^(2) = 1756.92, *p* < 0.001, and a significant interaction, *χ*^2^(2) = 22.38, *p* < 0.001. The probabilities were again higher for older than younger adults. The probabilities were also again significantly higher for the conditions with corrected fake news than the condition with affirmed real news, and for corrections of fake news that appeared thrice than once, smallest *z* ratio = 24.97, *p* < 0.001. The interaction showed that the higher probability for older than younger adults was greater for affirmations of real news, *z* ratio = 6.03, *p* < 0.001, than both corrections of fake news, largest *z* ratio = 2.82, *p* < 0.01. These results suggest that younger adults remembered corrections more accurately and that memory sensitivity was higher when fake news had appeared more often.

Signal detection analyses confirmed these sensitivity differences. The model for *d’* (Fig. 2A, bottom right panel) indicated significant effects of Age, *χ*^2^(1) = 3.98, *p* < 0.05, and Headline Type, *χ*^2^(1) = 16.53, *p* < 0.001, and no significant interaction, *χ*^2^(1) = 0.48, *p* = 0.49. Again, memory for topics being associated with corrections was more accurate for younger than older adults and for corrections of fake news that appeared thrice than one. The same model for *c* (Fig. 2B, bottom right panel) indicated significant effects of Age, *χ*^2^(1) = 25.24, *p* < 0.001, and Headline Type, *χ*^2^(1) = 16.58, *p* < 0.001, and no significant interaction, *χ*^2^(1) = 0.51, *p* = 0.47. Response bias was again more conservative for younger than older adults. These results replicate Experiment 1 in showing that more fake news exposure led to more accurate remembering that it was corrected. Such remembering was more precise for younger adults who also showed more conservative reporting of topics being corrected (for another example of such age differences in response bias using a free recall paradigm, see Huff et al., 2011).

### Overall cued recall: Phase 3

We examined the effects of fake news exposure prior to corrections on subsequent memory accuracy by assessing cued recall for real and fake news details in Phase 3. We assessed memory accuracy by comparing correct recall of real news details, intrusions of fake news details, and correct recall of fake news details. We used separate models with Age and Headline Type as fixed effects for each memory measure.

#### Correct recall of real news

Figure 3D displays correct recall of real news details, which refers to when participants recalled the real news detail that appeared in Phase 2. The model indicated no significant effect of Age, *χ*^2^(1) = 0.38, *p* = 0.54, a significant effect of Headline Type, *χ*^2^(2) = 258.78, *p* < 0.001, and no significant interaction, *χ*^2^(2) = 0.62, *p* = 0.73. Real news recall was better for affirmations of real news than corrections of fake news, smallest *z* ratio = 13.89, *p* < 0.001, and the latter did not differ based on fake news exposure, *z* ratio = 0.35, *p* = 0.94. As in Experiment 1, these results show that repeating real news improved memory, and suggest that the lack of fake news exposure effects reflected offsetting improvement and impairment that depended on detection of and memory for corrections.

#### Intrusions of fake news

Figure 3E displays intrusions of fake news, which refers to when participants reported Phase 1 fake news when trying to recall Phase 2 real news. Intrusions for affirmed real news were fake details that never appeared in the experiment. These extra-experimental intrusions come from semantic memory and provide baseline measures of knowledge and guessing. The model indicated no significant effect of Age, *χ*^2^(1) = 3.22, *p* = 0.07, a significant effect of Headline Type, *χ*^2^(2) = 256.14, *p* < 0.001, and no significant interaction, *χ*^2^(2) = 1.68, *p* = 0.43. Similar to false recognition in Experiment 1, intrusions were significantly higher for both types of corrected fake news than affirmed real news, smallest *z* ratio = 12.19, *p* < 0.001. In contrast to false recognition in Experiment 1, intrusions were significantly higher for thrice- than once-presented fake news, *z* ratio = 4.77, *p* < 0.001. Taken with Experiment 1, these results suggest that the impairment from more fake news exposure was greater when the test procedure placed higher demands on recollection by requiring self-generation.

#### Correct recall of fake news

Figure 3F displays correct recall of fake news, which refers to when participants recalled fake details after indicating that the topic was corrected in Phase 2. For the affirmed real news condition, these are instances when participants reported fake news details due to knowledge or guessing. The model indicated no significant effect of Age, *χ*^2^(1) = 0.32, *p* = 0.57, and a significant effect of Headline Type, *χ*^2^(2) = 1042.95, *p* < 0.001, and a significant interaction, *χ*^2^(2) = 13.73, *p* < 0.01. Both age groups recalled fake news significantly better when it appeared thrice than once, *z* ratio = 8.04, *p* < 0.001, and when it was corrected than when it did not appear, smallest *z* ratio = 28.57, *p* < 0.001. The interaction showed that older adults “recalled” more fake news details for affirmed real news, *z* ratio = 3.28, *p* < 0.01, which may reflect their greater knowledge of news content than younger adults (Brashier et al., 2017). In contrast, younger and older adults showed no significant difference in recall of fake news details between the correction conditions, largest *z* ratio = 0.41, *p* = 0.68. These patterns replicate the fake news exposure effects on recognition in Experiment 1.

### Cued recall in Phase 3 for corrections detected in Phase 2 conditionalized on fake news recall or remembering corrections in Phase 3

We followed the same approach as in Experiment 1 to determine if repeated fake news led to offsetting improvements and impairments that depended on detection of and memory for corrections. Namely, we focused the following analyses on instances when corrections were detected in Phase 2 in the corrected fake news conditions. We assumed that recalling fake news in Phase 3 reflected recollection of corrections because participants had to respond “yes” that a topic was corrected to receive the opportunity to recall fake news as such.

#### Correct recall of real news

We examined whether correct recall of real news depended on correct recall of fake news by conditionalizing real news recall on fake news recall when participants indicated a remembered correction (Fig. 4C). We used a model including Age, Fake News Recall, and Headline Type as fixed effects. Fake news recall had two levels: correct recall (fake news recalled; left panel) and incorrect recall (fake news not recalled; right panel).

Table 10 shows the model results. A significant effect of Fake News Recall, *χ*^2^(1) = 495.93, *p* < 0.001, indicated that real news recall was higher when fake news was recalled than when it was not. Also, a significant Age × Fake News Recall interaction, *χ*^2^(1) = 15.41, *p* < 0.001, showed that when fake news was recalled, real news recall was significantly higher for younger than older adults, *z* ratio = 4.24, *p* < 0.001; conversely, when fake news was not recalled, real news recall did not differ between age groups, *z* ratio = 0.19, *p* = 0.85. Further, a significant effect of Headline Type, *χ*^2^(1) = 11.75, *p* < 0.001, showed that real news recall was lower when fake news appeared thrice than once. Similar to Experiment 1, these results show that additional fake news exposure led to more trials on which real news recall received the benefits associated with fake news recall, but also impaired real news recall overall.

**Table 10:** Model results for correct recall of real news in Phase 3 for detected corrections in Phase 2 conditionalized on fake news recall in Phase 3: experiment 2

Effect	*χ*^2^	*df*	*p*
Age	5.34	1	= .02
Fake News Recall	495.93	1	< .001
Headline Type	11.75	1	< .001
Age × Fake News Recall	15.41	1	< .001
Age × Headline Type	< 0.01	1	= .99
Fake News Recall × Headline Type	2.38	1	= .12
Age × Fake News Recall × Headline Type	0.46	1	= .50

#### Intrusions of fake news

We next examined the dependence between intrusions of fake news and memory for corrections by conditionalizing such intrusions on remembering corrections (Fig. 4D). We used the same modeling approach as in the previous analyses. The two levels of remembering corrections were correct and incorrect classifications of corrected topics (Correction Remembered and Correction Not Remembered, respectively). We could not conditionalize these responses on fake news recall accuracy, as in the conditional analyses of correct real news recall, because the fake news details were redundant with the intrusions measured here.

Table 11 shows the model results. A significant effect of Remembering Corrections, *χ*^2^(1) = 219.23, *p* < 0.001, showed that intrusion rates were lower when corrections were remembered than when they were not. There was also a significant Age × Remembering Corrections interaction, *χ*^2^(1) = 7.71, *p* < 0.01. When corrections were remembered, intrusions of fake news were significantly lower for younger than older adults, *z* ratio = 4.21, *p* < 0.001; conversely, when corrections were not remembered, intrusions did not differ between age groups, *z* ratio = 0.70, *p* = 0.48. Additionally, a significant Remembering Corrections × Headline Type interaction, *χ*^2^(1) = 8.84, *p* < 0.01, showed that when corrections were remembered, intrusions did not differ based on fake news exposure, *z* ratio = 1.05, *p* = 0.29; conversely, when corrections were not remembered, intrusions were significantly higher for fake news that appeared thrice than once, *z* ratio = 4.85, *p* < 0.001. These patterns are somewhat parallel to those for correct real news recall in showing that more fake news exposure led to more trials with fewer errors when corrections were remembered (left panel, larger point sizes), but also led to larger magnitudes of errors when corrections were not remembered (right panel, higher point heights).

**Table 11:** Model results for intrusions of fake news in Phase 3 for detected corrections in Phase 2 conditionalized on remembering corrections in Phase 3: experiment 2

Effect	*χ*^2^	*df*	*p*
Age	10.01	1	< .01
Remembering Corrections	219.23	1	< .001
Headline Type	14.38	1	< .001
Age × Remembering Corrections	7.71	1	< .01
Age × Headline Type	0.29	1	= .59
Remembering Corrections × Headline Type	8.84	1	< .01
Age × Remembering Corrections × Headline Type	0.43	1	= .51

### Recollection and familiarity process estimates for Phase 3 cued recall

We used the same MPT model as in Experiment 1 to assess potential differences in the contributions of recollection and familiarity to headline recall. Table 7 (bottom section) shows that for recollection, there were no credible effects of Age or Headline Type, as in Experiment 1. However, in contrast to Experiment 1, for familiarity, younger adults showed credibly higher familiarity for fake news that appeared thrice than once (0.14 [0.05, 0.22]), whereas older adults show no credible difference (0.03 [-0.07, 0.12]). However, both groups showed lower familiarity estimates here than in Experiment 1, possibly due to the self-generation of cued recall, leading to low observation counts for correct real news recall and intrusions of fake news. This may have reduced the sensitivity to a fake news repetition effect in older adults or to an age effect in familiarity. The model fit of the covariances was not adequate for either group, so these results should be interpreted cautiously.

### Accuracy ratings for recalled headlines: Phase 3

We next examined how fake exposure affected belief accuracy for fake news correction headline types that participants recalled as real news in Phase 3. To assess belief accuracy, we compared accuracy ratings for correct recall of real news from Phase 2 and intrusions of fake news from Phase 1 (Fig. 5, right panel). As described earlier, belief accuracy was indicated by the extent to which accuracy ratings were higher for correct recall and lower for intrusions; larger differences indicated greater belief accuracy. A model with Age, Response Type, and Headline Type as fixed effects indicated a significant effect of Response Type, *χ*^2^(1) = 252.25, *p* < 0.001, showing higher accuracy ratings for correct recall of real news than intrusions of fake news. Table 12 shows that no other effects were significant. The absence of any other significant effects indicated that belief accuracy did not differ based on age group or fake news exposure.

**Table 12:** Model results for accuracy ratings in Phase 3: experiment 2

Effect	*χ*^2^	*df*	*p*
Age	2.59	1	= .11
Headline Type	3.52	1	= .06
Response Type	252.25	1	< .001
Age × Headline Type	0.19	1	= .66
Age × Response Type	3.55	1	= .06
Headline Type × Response Type	0.52	1	= .47
Age × Headline Type × Response Type	< .01	1	= .95

We further assessed belief accuracy by conducting conditional analyses of accuracy ratings (Fig. 6, bottom panels). Accuracy ratings for correct recall of real news were conditionalized on recall of fake news (left panel). The model included Age, Headline Type, and Fake News Recall as fixed effects. A significant effect of Age, *χ*^2^(1) = 4.98, *p* = 0.03, showed that accuracy ratings were higher overall for older than younger adults. Also, a significant effect of Fake News Recall, *χ*^2^(1) = 34.56, *p* < 0.001, showed that accuracy ratings were higher when fake news was recalled than when it was not. Table 13 (top section) shows that no other effects were significant. The absence of any other significant effects indicated that the improvement in belief accuracy associated with correct recall of fake news, shown by higher accuracy ratings, did not differ based on age group or fake news exposure.

**Table 13:** Model results for conditionalized accuracy ratings for correct recall of real news and intrusions of fake news in Phase 3: experiment 2

Response type	Effect	*χ*^2^	*df*	*p*
Correct recall of real news	Age	4.98	1	= .03
	Headline Type	0.66	1	= .42
	Fake News Recall	34.56	1	< .001
	Age × Headline Type	0.82	1	= .37
	Age × Fake News Recall	< .01	1	= .99
	Headline Type × Fake News Recall	0.04	1	= .84
	Age × Headline Type × Fake News Recall	< .01	1	= .95
Intrusions of fake news	Age	0.38	1	= .54
	Headline Type	1.24	1	= .27
	Remembering Corrections	10.60	1	< .01
	Age × Headline Type	0.03	1	= .85
	Age × Remembering Corrections	19.76	1	< .001
	Headline Type × Remembering Corrections	0.11	1	= .74
	Age × Headline Type × Remembering Corrections	0.72	1	= .40

Finally, accuracy ratings for intrusions of fake news were conditionalized on remembering corrections (right panel). The model included Age, Headline Type, and Remembering Corrections as fixed effects. There was a significant effect of Remembering Corrections, *χ*^2^(1) = 10.60, *p* < 0.01, and a significant Age × Remembering Corrections interaction, *χ*^2^(1) = 19.76, *p* < 0.001. For younger adults, accuracy ratings did not differ based on whether they remembered corrections, *t*(797) = 1.14, *p* = 0.25; conversely, for older adults, accuracy ratings for intrusions were significantly lower (and therefore more accurate) when they remembered corrections than when they did not, *t*(809) = 5.38, *p* < 0.001. Table 13 (bottom section) shows that no other effects were significant.

## Discussion

Experiment 2 showed that the lack of fake news exposure effects on overall correct recall of real news (Fig. 3D) reflected offsetting improvements and impairments that depended on recall of fake news (Fig. 4C), similar to the findings for recognition of real news headlines in Experiment 1. In contrast to Experiment 1, additional fake news exposure led to more intrusions of fake news (Fig. 3E) because the offsetting effects associated with remembering correction were less balanced (Fig. 4D). As in Experiment 1, repeating fake news improved sensitivity to detecting corrections in Phase 2 (Fig. 2A), remembering corrections in Phase 3 (Fig. 2A), and recall of fake news in Phase 3 (Fig. 3F). These memory outcomes were associated with comparable benefits to accurate recall of headlines, comprising increased correct recall of real news and decreased intrusions of fake news (Fig. 4C, D). In contrast to Experiment 1, these memory benefits were greater for younger than older adults. However, as in Experiment 1, when fake news was not recalled and corrections were not remembered in Phase 3, more fake news exposure impaired recall accuracy to a greater extent (Fig. 4C, D). Finally, as in Experiment 1, younger adults remembered corrections more precisely and classified headline topics as corrected more conservatively (Fig. 2A, B).

The findings from Experiment 2 are generally consistent with the findings in Experiment 1 and work showing that benefits to real news recall of detecting corrections require subsequent recall of fake news (Kemp et al., 2022b). The offsetting improvements and impairments to real news recall accuracy that depended on fake new recall and remembering corrections provided further support for the integrative encoding view (Ecker et al., 2017; Wahlheim et al., 2020), and again supported the suggestion that familiarity-based memory errors occur in the absence of recollection (cf. Skurnik et al., 2007). The finding that older adults’ real news recall accuracy benefitted less when fake news was recalled and corrections were remembered suggests that older adults engaged integrative encoding and subsequent recollection of associations less effectively. This is consistent with studies showing age-related associative memory deficits (for a review, see Park & Festini, 2017). Although this potential age-related difference was not borne out in the MPT parameter estimates (Table 7, bottom section), those estimates did reveal a key difference between experiments. The cued recall task here appeared to rely more heavily on recollection than familiarity, whereas the reverse was true for the recognition task in Experiment 1. This difference may explain why more exposure to fake news here results in more intrusions. However, this possibility requires further validation because model fitting challenges warrant cautious interpretation.

Finally, belief accuracy in Phase 3, indicated by higher accuracy ratings for correct real news recall than intrusions of fake news, did not differ across fake news exposures and age groups (Fig. 5, right panel). This finding replicated the pattern in recognition memory from Experiment 1. Also similar to Experiment 1, recalling fake news was associated with greater belief accuracy in the form of higher accuracy ratings for recalled real news (Fig. 6, bottom left panel); this benefit did not differ between age groups. However, older adults showed greater belief accuracy for intrusions when corrections were remembered, whereas younger adults showed no difference in belief accuracy based on memory for corrections (Fig. 6, lower right panel). Collectively, these results suggest that both age groups based their beliefs partly on memory for corrections. Their reliance on memory to determine the accuracy of retrieved details depended on response type, as in Experiment 1, but not fake news exposure, inconsistent with Experiment 1. These disparities indicate that the different retrievals evoked by recognition and cued recall, perhaps reflecting differences in recollected content, determine which memory cues can be accessed to identify headline veracity.

### General discussion

Two experiments characterized the effects of correcting repeated fake news on the accuracy of memory for and beliefs in news details in younger and older adults. Both age groups showed consistent patterns of overall memory accuracy in recognition and cued recall, but fake news repetition effects varied across measures (Fig. 3). Fake news repetition did not affect overall recognition or recall of real news or false recognition of fake news; but such repetition increased correct recognition, correct recall, and intrusions of fake news. The lack of overall fake news repetition effects reflected improvements and impairments that depended on how often participants remembered corrections as such and the corresponding fake news details (Fig. 4). Retrieving fake news details and remembering corrections were both associated with improved memory accuracy. The magnitude of this improvement did not differ based on fake news exposure. However, more fake news exposure led to higher rates of correctly retrieving fake news headline details and, thus, more instances of improved correct retrieval of real news details. Conversely, failure to retrieve fake news details and remember corrections were both associated with impaired memory accuracy that was greater after more fake news exposures. The improved cued recall accuracy associated with recalling fake news and remembering corrections was greater for younger than older adults. This suggests that younger adults had more precise recollection of associations between real and fake news.

Both age groups indicated that correctly retrieved real news details were more accurate than incorrectly retrieved fake news details (Fig. 5). This belief accuracy did not differ based on age or fake news exposure. Belief accuracy for correctly retrieved real news details was improved when fake news details were retrieved (Fig. 6). This improvement was slightly greater for younger than older adults in recognition but did not differ between age groups in cued recall. A more complex picture emerged for beliefs in falsely retrieved fake news details. Remembering corrections was associated with lower accuracy ratings for falsely recognized fake news after three exposures to fake news for both age groups. Also, remembering corrections was associated with lower accuracy ratings for intrusions of fake news for older but not younger adults. Collectively, these findings underscore the interplay of age, memory, and beliefs and emphasize the need to consider how retrieval requirements affect memory precision in the service of assessing the veracity of retrieved details.

The present findings have implications for the controversy regarding the effects of repeating misinformation with corrections on subsequent memory, beliefs, and reasoning. Two prominent views have been proposed to explain these effects. The familiarity-backfire view proposes that repeating misinformation may reduce correction efficacy by increasing its familiarity and processing fluency (Autry & Duarte, 2021; Schwarz & Jalbert, 2020). Conversely, the integrative-encoding view proposes that repeating fake news may enhance correction effectiveness by promoting conflict saliency and the co-activation of both the misinformation and its correction (Ecker et al., 2017). Both views are compatible with a dual-process perspective, which emphasizes that memory accuracy for corrected information varies depending on whether retrieval is familiarity-based, relying on a general feeling of memory strength, or recollection-based, involving the retrieval of contextual details, which may include associations between true and false information (e.g., Wahlheim et al., 2020).

Our study provided evidence that is more compatible with the integrative encoding than familiarity-backfire view. The frequency and degree to which recollection of earlier-detected corrections counteracted familiarity-based misattributions of fake news depended on fake news exposures. Repeating fake news provided more opportunities to improve memory accuracy by increasing the number of trials on which detected corrections and fake news details were later recollected. Moreover, repeated fake news exposure impeded memory more when earlier-detected corrections and fake news details were not later recollected. Taken with the absence of fake news exposure effects on overall recall of real news, these findings suggest that more accessible fake news promoted recollection of integrated representations including associations between true and false information. However, the more accessible fake news also increased errors when corrections were not recollected. This combination cannot be explained by the familiarity-backfire view because, although it predicts memory impairment from more fake news exposure, it does not predict the mixture of effects that depended on the success of recollecting corrections. This mixture of fake news exposure effects corresponds with research showing that fake news reminders improve memory for corrections and recollection that fake news was corrected (Kemp et al., 2022a, 2022b; Wahlheim et al., 2020). The present findings are also more generally consistent with the Memory-For-Change framework (Jacoby et al., 2015; Wahlheim & Jacoby, 2013), which proposes that retrieving outdated information while encoding similar but not identical stimuli can improve memory when the changes are recollected but impair memory when they are not.

The present study also addressed the mechanisms underlying potential age-related differences in repeated fake news exposure effects on memory accuracy. According to dual-process theories, aging may impair recollection of contextual details, making older adults more reliant on familiarity during retrieval (Jacoby, 1999; Jennings & Jacoby, 1993). This leads to the prediction that the increase in familiarity resulting from repeated exposure to fake news should disproportionately impair memory accuracy for older adults. In contrast to this prediction, overall memory accuracy was comparable for both groups in most situations. However, older adults consistently remembered which topics included corrected fake news less precisely than younger adults, with younger adults showing more conservative response biases. Older adults may have been more willing to identify topics as corrected using cues other than recollection-based retrieval. Additionally, when participants recalled fake news details and remembered corrections, the associated benefits to real news recall were smaller for older than younger adults. This finding suggests that older adults integrated real and fake news details less well than younger adults, leading to poorer recollection of corrections, consistent with earlier studies (Wahlheim, 2014; Wahlheim & Zacks, 2019). However, this age difference was not observed for recognition memory suggesting that the environmental support repaired this deficit. Although there were slight age differences in specific memory measures, those differences were too subtle to be observed on the overall memory measures. Older adults’ relatively preserved memory accuracy may reflect support from intact semantic memory for the topics reported in news headlines (cf. Park et al., 2002). However, we cannot rule out the possibility that these groups of older adults were the most motivated and capable of their cohort.

One noteworthy finding was that increased exposure to fake news did not affect overall recognition or recall of real news details. This finding contrasts with studies using narrative-based paradigms showing that repeating misinformation increased its contribution to inferential reasoning compared to a single exposure (Ecker et al., 2011). However, the current finding is consistent with memory studies showing that more repetitions of competing paired associates does not increase proactive interference because of the offsetting effects it creates (e.g., Wahlheim & Jacoby, 2013). This discrepancy across literatures may reflect differences in how stimulus features and repetitions provide opportunities for integrative encoding. For example, repeated exposure to word pairs that overlap perceptually and conceptually with pairs including shared and distinctive features (e.g., music-song, music-sing) may increase integrative encoding enough to offset proactive interference. In contrast, sentences in narrative-based paradigms may not always include the same high degree of overlap. Future research could explore correction efficacy on subsequent memory across a broader range of fake news repetitions, stimulus features, and even examine interactions with various retention intervals. The latter is crucial because, in everyday life, information learned from news headlines on social media may not be relevant until weeks or months later. Over these longer time periods, the ability to use recollection to oppose fake news familiarity erodes. Our goal here was to test participants after delays that provide theoretically appropriate conditions for examining repetition effects on familiarity-based memory errors. However, the present retention intervals may still have been too short to reveal age-related differences in the interference created by more fake news repetitions.

The present study also contributes to the literature on the interplay between memory and beliefs in correcting misinformation. Previous research in this area mainly focused on correction effects on inferential reasoning with less emphasis on the role of memory (for a review, see Lewandowsky et al., 2012). However, memory has routinely been shown to shape beliefs (Begg et al., 1992; Berinsky, 2017; Kowalski & Taylor, 2017; Newman et al., 2022; Swire-Thompson et al., 2023; but see, Collier et al., 2023). Our work using fake news correction paradigms showed that memory for corrections was associated with more accurate beliefs—defined as the extent to which accuracy ratings for correct recall of real news details was higher than for intrusions of fake news details—especially for corrections with veracity labels (Kemp et al., 2022b; Wahlheim et al., 2020). The present findings replicate and extend that work, showing that retrieving fake news was associated with greater belief accuracy for retrieved real news, regardless of fake news exposures. These benefits were slightly greater for younger than older adults in recognition, but not cued recall. These findings suggest that the quality of mnemonic content can drive perceptions of headline veracity. Recollecting fake news (and that it was corrected) and using that information to rate the accuracy of retrieved details may have led participants to be more certain about the veracity of retrieved real news.

In contrast to the mostly consistent conditional belief accuracy for retrieved real news, the patterns for false recognition and intrusions of fake news varied across experiments. In Experiment 1, remembering corrections was only associated with lower accuracy ratings for false recognition of thrice-presented fake news. In Experiment 2, remembering corrections was only associated with lower accuracy ratings for intrusions of fake news for older adults, regardless of fake news exposure. Although the mechanisms underlying the improvements in belief accuracy are unclear, we offer some possibilities to consider. Participants may only base their accuracy ratings on memory for corrections during false recognition when correction episodes are sufficiently recollected. This may be required to overcome the otherwise similar feelings of familiarity evoked by response alternatives because they are present in the environment. Additionally, when self-generating responses, younger adults may engage in more conservative reporting than older adults. Consequently, corrections may better distinguish between intrusions reported with varying levels of certainty for older adults. In contrast, younger adults’ certainty in the accuracy of reported intrusions may be more homogeneous. Whatever the mechanisms may be, these findings clearly indicate that mnemonic variables such as retrieval demands and the quality of retrieved information interact with beliefs in the accuracy of retrieved details.

### Limitations

The present study had several limitations. First, we used an online platform for broad recruitment across the USA, but time constraints limited our ability to characterize participants’ cognitive abilities. The online recruitment method may have attracted more motivated and capable older adults (Ryan & Campbell, 2021). This raises uncertainty about the sample’s representativeness, as Prolific tends to attract healthy, technologically proficient, and well-educated people (Turner et al., 2020). We may have observed typical age-related memory deficits (e.g., Jacoby, 1999) from a diverse community sample. Nonetheless, online methods hold value in paralleling daily news engagement, thereby enhancing generalizability.

Second, the MPT model for cued recall in Experiment 2 did not adequately fit the covariances. Consequently, we could not confidently interpret the parameter estimates. Also, our model choice was tailored to test assumptions within one framework, though others could have been considered. We opted for a dual-process model given its suitability for assessing recollection and familiarity estimates, consistent with our prior work (Kemp et al., 2022b). Unexpectedly, overall memory performance and MPT results did not show age-related susceptibility to memory errors after repeated fake news exposure, which runs counter to the view that older adults have impaired recollection. While we cannot explain this, future studies could modify the task design while comparing fits across other feasible models to seek stronger model-based evidence for the process assumptions that we made here.

Third, although some patterns of memory and belief accuracy replicated across experiments, others did not. This likely reflects differences in how recognition memory and cued recall tasks evoke retrievals. We assumed that any differences across experiments could be attributed to recognition providing more environmental support and cued recall requiring more recollective self-generated retrieval. However, more conclusive evidence for retrieval demands being the primary driving factor requires future replication attempts and within-participant comparisons.

Lastly, we examined associations between memory and beliefs for headlines post-correction using a less typical measure of accuracy ratings. Participants attempted to retrieve real news details and then rated the accuracy of the retrieved details. This sequence could have biased participants to report what they strongly believe is true, which would restrict the range of accuracy ratings to values above a reporting threshold. However, this procedure has revealed variability in accuracy ratings for retrieved details (Kemp et al., 2022b), even when participants were told to withhold reporting details that they believe are false (Wahlheim et al., 2020; Experiment 2). We propose that the present accuracy rating measures capture variability in the extent to which participants retrieve details diagnostic of headline veracity akin to how people make retrospective metacognitive assessments of the accuracy of retrieved content.

Future research should examine memory and belief associations for headlines post-corrections using various methods. For example, future studies could include retrospective confidence judgments of both memory and accuracy ratings (cf. Dobbs et al., 2023) to more precisely characterize the subjective experiences created by different correction types. Future studies could also contrast correction effects on memory and beliefs from the current "memory-first" paradigm with effects shown in standard belief updating paradigms. In the latter, participants rate the accuracy of all items, regardless of whether they could have retrieved the details (Swire-Thompson et al., 2023; Wahlheim et al., 2024). This approach addresses the concern that beliefs are misestimated by the current method because participants only rate the accuracy of subsets retrieved details.

## Concluding remarks

Our study contributes to the literature on the efficacy of corrections after repeated exposure to fake news. Neither age nor fake news repetitions affected overall recognition or cued recall of real news or false alarms to fake news, but repeated fake news was both better recalled and intruded more in cued recall. Repeating fake news created more instances when memory benefitted from detecting and remembering corrections, but doing so also led to greater impairment when detected corrections were not remembered. Although there were minimal age differences across measures, older adults were less discerning in their identification of retrieved corrections and showed less evidence of preserved integration of real and fake news details. Finally, the qualities of retrieved details may have determined how participants used memory for corrections to evaluate the veracity of those details, but future studies are needed. Collectively, the findings highlight the need for interventions aimed at changing beliefs and actions to consider the role of memory and its potential influence on everyday decision-making.

## Data Availability

The stimuli, data, and analysis scripts for both experiments are publicly available on the Open Science Framework: https://osf.io/vqwtu/.

## Footnotes

1 After removing items that received a “6” familiarity rating in Phase 1, the model showed a significant Age × Response Type interaction, *χ*^2^(1) = 4.19, *p* = .04. Accuracy ratings for false recognition of fake news were significantly higher for older than younger adults, *z* ratio = 2.47 *p* = .01, but accuracy ratings for correctly recognized real news did not differ between age groups, *z* ratio = 1.10, *p* < .27.
